# New insights into the genetic composition and phylogenetic relationship of wolves and dogs in the Iberian Peninsula

**DOI:** 10.1002/ece3.2949

**Published:** 2017-05-11

**Authors:** Ana Elisabete Pires, Isabel R. Amorim, Carla Borges, Fernanda Simões, Tatiana Teixeira, Andreia Quaresma, Francisco Petrucci‐Fonseca, José Matos

**Affiliations:** ^1^ Biotechnology and Genetic Resources UnitNational Institute of Agrarian and Veterinary Research, I.P. (INIAV)OeirasPortugal; ^2^ Centre for Ecology, Evolution and Environmental Changes (cE3c)Faculty of SciencesUniversity of LisbonLisbonPortugal; ^3^ Centre for Ecology, Evolution and Environmental Changes/Azorean Biodiversity Group and Universidade dos AçoresFaculdade de Ciências Agrárias e do AmbienteAçoresPortugal

**Keywords:** genetic differentiation, genetic diversity, Iberian *Canis*, mitochondrial DNA, Y chromosome

## Abstract

This study investigates the gene pool of Portuguese autochthonous dog breeds and their wild counterpart, the Iberian wolf subspecies (*Canis lupus signatus*), using standard molecular markers. A combination of paternal and maternal molecular markers was used to investigate the genetic composition, genetic differentiation and genetic relationship of native Portuguese dogs and the Iberian wolf. A total of 196 unrelated dogs, including breed and village dogs from Portugal, and other dogs from Spain and North Africa, and 56 Iberian wolves (wild and captive) were analyzed for nuclear markers, namely Y chromosome SNPs, Y chromosome STR loci, autosomal STR loci, and a mitochondrial fragment of the control region I. Our data reveal new variants for the molecular markers and confirm significant genetic differentiation between Iberian wolf and native domestic dogs from Portugal. Based on our sampling, no signs of recent introgression between the two subspecies were detected. Y chromosome data do not reveal genetic differentiation among the analyzed dog breeds, suggesting they share the same patrilineal origin. Moreover, the genetic distinctiveness of the Iberian wolf from other wolf populations is further confirmed with the description of new mtDNA variants for this endemism. Our research also discloses new molecular markers for wolf and dog subspecies assignment, which might become particularly relevant in the case of forensic or noninvasive genetic studies. The Iberian wolf represents a relic of the once widespread wolf population in Europe and our study reveals that it is a reservoir of unique genetic diversity of the grey wolf, *Canis lupus*. These results stress the need for conservation plans that will guarantee the sustainability of this threatened top predator in Iberia.

## 1 Introduction

The Iberian grey wolf subspecies, *Canis lupus signatus* (Cabrera, 1907) and the domestic dog, *Canis lupus familiaris*, coexist in the Iberian Peninsula. The domestic dog has existed in this territory at least since the Upper Paleolithic (Detry & Cardoso, 2010; Pionnier‐Capitan et al., 2011), and extant dog breeds native to Iberia show a wide variety of forms and functions.

Regarding the Iberian wolf, the latest estimates suggest that approximately 2,400 wolves exist in the wild in Iberia, as a result of population expansion after a population minimum due to human activities in the early 20th century (Álvares, 2011). Although management and conservation unit status are recognized for this wolf subspecies (Chapron et al., 2014; Pimenta et al., 2005), different policies are in place in Portugal and Spain. In Portugal, where the last census estimated a population size of only 300 individuals (Pimenta et al., 2005) and the current trend is unknown (Torres & Fonseca, 2016), the conservation status of the Iberian wolf is “Endangered,” and it is fully protected by law (Portuguese Law 90/88). In Spain, the Iberian wolf conservation status is “Vulnerable” and its protection varies regionally. In some areas, it is fully protected and in others, such as northwestern Spain, hunting is legal. Most wolves occur in the northwestern part of the Iberian Peninsula and south of the Douro river, with an uncertain small group in Sierra Morena (south of Spain) (Álvares, 2011; López‐Bao et al., 2015).

In the Iberian Peninsula, both dog and wolf subspecies have been the focus of several studies where molecular markers were used to describe genetic composition, population structure, and phylogeny. Microsatellite and AFLP analyses of modern dog suggest that the genetic variability of dog breeds is structured according to breeds and geography (Pires et al., 2009) and that Portuguese dogs have multiple mtDNA haplotypes in agreement with the mtDNA diversity found in other European dogs (Pires et al., 2006).

The occurrence of gene flow between domestic dogs and Iberian wolves has been documented and estimated (van Asch et al., 2010; Fan et al., 2016; Godinho et al., 2011, 2015; Ramirez et al., 2006). However, wolf–dog hybrids in Iberia have only been reported for marginal and recently expanded wolf nuclei (Godinho et al., 2011) with the possible involvement of hunting or livestock guard dogs, rather than feral dogs. Another study in the Caucasus region reports the existence of gene flow between wolves and livestock guard dogs (Kopaliani et al., 2014).

Information from other genetic markers, such as mitochondrial DNA (mtDNA) or genomewide SNPs, is also available for Iberian wolves (van Asch et al., 2005; Godinho & Ferrand, 2007; Godinho et al., 2011; Pilot et al., 2010, 2014; Vilà et al., 1997, 1999). Briefly, these latter studies concluded that for *Canis* found in Iberia, particularly in Portugal: (1) Iberian wolves exhibit both unique mtDNA haplotypes and haplotypes that are shared with other European and Asian wolves; (2) some wolf haplotypes are widespread, while others are rare with a restricted distribution, but there is no obvious mtDNA haplotype geographic structure; (3) dog and wolf mtDNA haplotypes are mostly distinct, with few haplotypes shared; and (4) Iberian wolf genome analysis shows signatures of long‐term isolation from other European wolf populations, evidence of diversifying selection due to local adaptation and admixture with dogs.

High‐resolution information combining Y chromosome STR and SNP markers for dogs and wolves from the Iberian Peninsula has not, however, been disclosed. Y‐specific molecular markers are important to complement mtDNA studies and have proven useful to investigate male‐mediated evolutionary patterns in both wild and domesticated species (*e.g.,* Ginja, Telo da Gama, & Penedo, 2009; Ginja et al., 2010; Godinho et al., 2011; Gomerčić, Sindičić, & Florijančić, 2013; Götherström et al., 2005; Hellborg, 2004; Meadows et al., 2006; Petit, Balloux, & Excoffier, 2002; Schregel et al., 2015; Sundqvist et al., 2006).

For *Canis*, the most recent studies using Y chromosome markers are from Brown et al. (2011), Godinho et al. (2011), Ding et al. (2012), Gomerčić et al. (2013) and Sacks et al. (2013). Brown et al. (2011) studied Y‐STRs and Y‐SNPs in a large population of 633 worldwide dogs (including village and breed dogs) and concluded that modern European dog breeds possess roots in southeastern Asian dogs and that African dogs have different patrilineal origins. Godinho et al. (2011) used six Y‐STR loci in their study of Iberian wolf–dog hybridization and found six different Y‐linked haplotypes in 99 male wolves, and 14 different Y haplotypes in 78 male dogs (including breed and feral dogs). To further investigate the phylogeography of the domestic dog, Ding et al. (2012) studied a 14,437 base pairs (bp) sequence fragment of Y chromosomal DNA in 151 dogs distributed worldwide, which revealed 28 haplotypes distributed across five haplogroups. Additionally, they observed that a region in Asia, south of the Yangtze River (ASY), has nearly the full range of genetic diversity supporting previous evidence from mtDNA (Pang et al., 2009) that ASY was the main region where wolves were domesticated. Gomerčić et al. (2013) also used Y chromosome sequences and concluded that dogs and gray wolves from Croatia could not be differentiated as they shared a single haplotype. Sacks et al. (2013) used dog patrilines, by combining rapidly mutating Y‐STRs and slower mutating Y‐SNPs, to date the origin of modern western dogs.

As referred by Lescureux and Linnell (2014), more studies are needed to clarify the relationship between wolves and dogs. In line with this need, our research aims to analyze the overall genetic variability and the phylogenetic relationship between wolves and native dogs from the Iberian Peninsula. Our main objectives were as follows: (1) to capture unreported genetic variants for the Portuguese *Canis* populations; (2) to understand the strength of genetic divergence between paternal lineages of Portuguese native dogs and the Iberian wolf; (3) to investigate whether Iberian dogs and wolves represent genetically distinct reservoirs from their worldwide counterparts; and (4) to provide data to assist the conservation of the locally adapted Iberian wolf subspecies.

## 2 Materials and Methods

### 2.1 Sample collection and DNA extraction

#### 2.1.1 Dogs

A total of 168 breed dog samples (76 males and 92 females) were selected based on breed standards and avoiding related animals back to the third generation. Blood, hairs or tissue samples were collected for nine Portuguese native dog breeds at dog shows, breeding kennels, and distinct locations in historical breed regions, and for other breeds in such geographically neighboring regions as Spain and Northwest Africa (Morocco) (Table 1). Samples were collected by a veterinarian and in the presence of the owners. Additionally, samples were collected from live village dogs held in several shelters in Portugal (Azores, Estrela Mountain, and Alentejo) and Tunisia (northwest Africa). Twenty‐eight village dog samples (10 males and 18 females) were collected from specimens whose phenotypes could not be assigned to any breed, including common worldwide breeds, nor resembled the phenotype of wolf–dog hybrids (Table 1).

**Table 1:** *Canis lupus familiaris* sampling. Dog samples (*N* = 196, 86 males, 110 females) are organized by breed affiliation and function, as recognized by the World Canine Organization (Fédération Cynologique Internationale, FCI)

Dog breed groups (sample size)	Designation of breed/population of dogs	Sample size (Y‐Chromosome and/or mtDNA)	Geographic location
Livestock guard dogs/Group 2 (60)	CLWD	9♂;8♀	NW Portugal
	EMD	10♂;12♀	East Portugal
	AM	8♂;9♀	SE Portugal
	TM	4♂;0♀	NE Portugal
Livestock herding dogs/Group 1 (29)	ACD	7♂;13♀	Azores archipelago
	PSD	4♂;5♀	SE Portugal
Fishing dogs/Group 8 (11)	PWD	7♂;4♀	South Portugal
Hunting dogs/Group 5 and 7 (38)	PP	3♂;6♀	Undetermined
	PWH	7♂;22♀	Undetermined
Other dog populations (39)	SM	7♂;3♀	Spain
	Aidi	6♂; 4♀	Morocco
	Sloughi	4♂;6♀	Morocco
	Tunisia village dogs	2♂;7♀	Tunisia
Portuguese Village dogs (19)	Portuguese village dogs	8♂;11♀	NE, SE mainland Portugal and Azores
Total (196)		86♂;110♀	
Individuals sampled per population (Average ± *SD*)		14 ± 6.8	

Breed/Dog Populations acronyms: ACD, Azores cattle dog, AM, Alentejo Mastiff (previously denominated Alentejo Shepherd dog), CLWD, Castro Laboreiro Watchdog, EMD, Estrela Mountain Dog, PP, Portuguese Pointer, PSD, Portuguese Sheepdog, PWD Portuguese Water Dog, PWH, Portuguese Warren Hound; SM, Spanish Mastiff, TM, Transmontano Mastiff, PortVilldog, Portuguese village dogs.

#### 2.1.2 Iberian wolves

**Figure 1:**
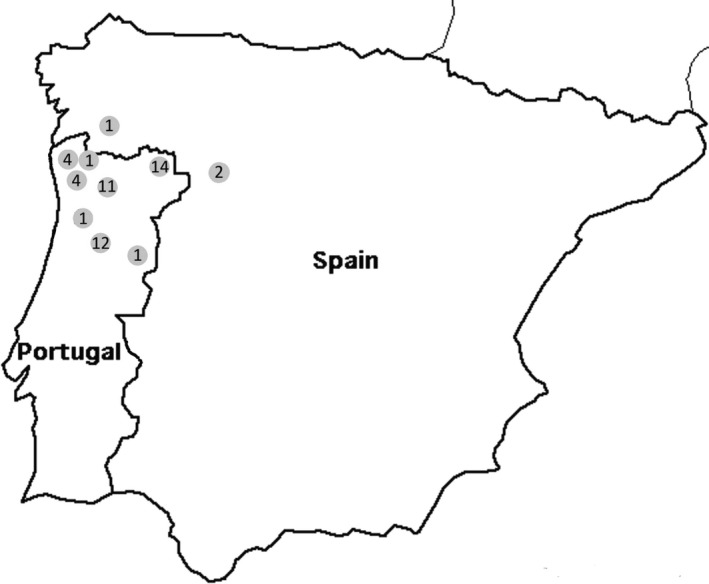
*Canis lupus signatus* sampling (*N* = 56, 44 males, 11 females, 1 undetermined sex). Geographic origin of Iberian wolf samples is mapped per region (number of samples per site are indicated inside dots) except for five individuals for which only country of origin is known (Portugal *N* = 4, Spain *N* = 1)

Wolf sampling included 56 individuals: 44 randomly collected wild dead animals from Portugal (*n* = 42) and Spain (*n* = 2); and 12 live captive specimens kept at the Iberian Wolf Recovery Centre (three specimens originally from Spain and nine from Portugal). Wild specimens' samples were either tissue or blood (from necropsied animals), obtained from the wolf tissue bank (SMLM) managed by the Portuguese Institute for the Conservation of Nature and Forest **(ICNF); blood from live captive animals was collected by a veterinarian.** Samples included 11 females, 44 males, and one specimen of undetermined sex and were collected between 1987 and 2008 (Figure 1).

Total genomic DNA was extracted from whole blood and tissue using a standard proteinase *K*/phenol–chloroform protocol (Sambrook, Fritsch, & Maniatis, 1989), the Nucleospin Blood QuickPure kit (Macherey‐Nagel), or a high salt method (Montgomery & Sise, 1990). DNA was extracted from hair roots in a 20% Chelex solution (Walsh, Metzger, & Higuchi, 1991) in a different DNA extraction dedicated room to avoid contamination.

### 2.2 Genetic markers

#### 2.2.1 Autosomal STR genotyping

Nineteen canid autosomal microsatellite markers (STRs) were genotyped in a set of 122 dogs and 52 wolves: AHT121 (Holmes et al., 2009); C22.279, CXX.109, CXX.173, CXX.225, FH2001, FH2054, FH2010 and FH2159 (Francisco et al., 1996); FH2247 (Richman et al., 2001); FH2611 (Eichmann, Berger, & Parson, 2006); FH2361 (Mellersh et al., 1997); FH4012 and FH3210 (Guyon et al., 2003); PEZ06 and PEZ08 (Neff et al., 1999); REN247M23 (Moore & Sacks, 2010); VWF.X (Shibuya et al., 1994); and C38 (van Asch et al., 2010). All forward primers were fluorescently labeled (6‐FAM, Hex, or NED from Applied Biosystems, Foster City, CA, USA). PCR and electrophoresis conditions are described in Supplementary Materials.

#### 2.2.2 Y chromosome SNPs

##### 2.2.2.1 Primer design

Sequences of newly designed primers for PCR amplification of Y chromosome fragments (Natanaelsson et al., 2006) and for SNaPshot extension reactions are presented in Tables S1 and S2, respectively. Primers for Ydog_20, Ydog_21, Ydog_28, Ydog_29, Ydog_30, Ydog_B, Ydog_G, and Ydog_N fragments were designed using the software Primer3 (http://simgene.com/Primer3) and based on GenBank sequences (Accession numbers: DQ973626‐DQ973805). The haplotype 1 sequence from isolate y20 from *Canis lupus familiaris* was used as reference for SNaPshot primer design (GenBank sequences: DQ973626, DQ973627, DQ973631, DQ973635, DQ973636, DQ973638, DQ973639 and DQ973642). PCR product sizes ranged from 130 to 492 bp (Table S1). Extension primers for SNaPshot reactions anneal immediately adjacent to the SNP site of interest on either the sense or antisense DNA strand (Table S2). To test for possible repetitive annealing targets, primer sequences were tested by previously aligning them against the NCBI sequence databases using the BLAST program.

For amplicons containing multiple SNPs, extension primers were designed to detect polymorphisms at nucleotide sites (nt) 599, 619, and 873 at the amplicon Ydog_28 (SNPs positions here named Ydog_28A, Ydog_28B, and Ydog_28C, respectively) and nt 66 and 146 at the amplicon Ydog_G (part 1) (SNPs positions here named Ydog_G1A and Ydog_G1B, respectively) according to the labeling scheme of Natanaelsson et al. (2006) (Table S2).

##### 2.2.2.2 PCR amplification

PCR amplifications were carried out individually for each Y chromosome fragment using the primer pairs described in Table S1. All reaction mixtures contained 25–50 ng of genomic DNA, 1× Biomix PCR master mix (Bioline), and 0.2 μmol/L of each primer in a total volume of 15 μL. PCR and electrophoresis conditions are described in Supplementary Materials. Six female samples from both subspecies were also PCR‐tested for all loci to check the Y‐specificity of the primer pairs.

##### 2.2.2.3 SNaPshot reaction for probe primer extension

This step consisted of a multiplex reaction for single‐base primer extension using the ABI PRISM^®^ SNaPshot^™^ Multiplex Kit (Applied Biosystems) according to the manufacturer's protocol. Two separate multiplex reactions were prepared: one included pooled PCR products and extension primers for loci Ydog_21, Ydog_28, Ydog_G (part 1) and Ydog_B (part 2) (SNPs position here named Ydog_B), covering seven dog‐specific SNPs; and the other multiplex included the PCR products and extension primers for the remaining loci mentioned in Table 2 (Ydog_20, Ydog_29, Ydog_30, Ydog_N), covering four SNPs.

**Table 2:** List of the 11 Y chromosome sequences analyzed and nucleotides detected in each polymorphic position for each subspecies. Fragments and nucleotides that separate domestic dogs from Iberian wolves are highlighted in bold

Y‐ chromosome fragment	Iberian and North African dogs' SNPs	Iberian wolves' SNPs
Ydog_20	A	A
Ydog_21	G	A/G
**Ydog_28A**	**A**	**G**
**Ydog_28B**	**A**	**C**
Ydog_28C	G	G/A
Ydog_29	A	A
Ydog_30	C	C
**Ydog_B**	**C**	**T**
Ydog_G1A	T	T
**Ydog_G1B**	**C**	**A**
Ydog_N	C	C
	Iberian and North African Dog haplogroup (*IbAfrDog HG*)	Iberian Wolf haplogroup (*IbWolf HG*)

##### 2.2.2.4 Y chromosome DNA sequence analysis

In the search for new polymorphisms, sequencing reactions were performed for each locus in dogs and wolves. PCR products were sequenced on both directions by BigDye Terminator v1.1 Sequencing Kit and analyzed on an automated fluorescence‐based ABI PRISM 3130 Genetic Analyser (Applied Biosystems).

##### 2.2.2.5 Y chromosome microsatellites

Four Y chromosome microsatellite (short tandem repeats, STRs), loci—990‐35, MS41A, MS41B, and MS34TTR (Bannasch et al., 2005), were analyzed. All loci were amplified in a single multiplex reaction using the primers presented in Table S3. PCR and electrophoresis conditions are described in Supplementary Materials.

#### 2.2.3 Mitochondrial DNA PCR amplification and sequencing

The primers ThrL 5′‐ GAA TTC CCC GGT CTT GTA AAC C ‐3′ and DLH‐can 5′‐ CCT GAG GTA AGA ACC AGA TG ‐3′ (Hailer & Leonard, 2008) were used to PCR amplify a 420‐bp mtDNA fragment from Iberian wolf samples, including the 3′ end of the tRNA‐Thr and part of the control region I. No sequences were generated for dog samples from Portugal as those are already available at GenBank (Pires et al., 2006). PCR conditions are described in Supplementary Materials.

Sequencing reactions were performed using the ABI PRISM BigDye Terminator chemistry and separated by electrophoresis on an automated fluorescence‐based ABI PRISM 3130 Genetic Analyzer (Applied Biosystems).

### 2.3 Statistical analysis

#### 2.3.1 Autosomal STRs data analysis

Standard measures of genetic diversity and the *F*_ST_ value were calculated in GenAlEx 6.5b (Peakall & Smouse, 2006) using a set of 122 dogs and 52 wolf genotypes. Genetic differentiation was further investigated using a Bayesian clustering procedure included in the STRUCTURE software version 2.3 (Falush, Stephens, & Pritchard, 2003; Pritchard, Stephens, & Donnelly, 2000). Five independent runs [*K* = 2; 100,000 Markov Chain Monte Carlo (MCMC) iterations; burn‐in period of 10,000 interactions] were set to run using the ‘admixture’ model and assuming that allele frequencies are correlated among populations for a *K* = 2. The STRUCTURE PLOT software (Ramasamy et al., 2014) was used to construct the STRUCTURE bar plot.

#### 2.3.2 Y chromosome markers

Regarding Y‐STRs, we estimated haplotype diversities and analyzed molecular variance (AMOVA) using GenAlEx v6.501 (http://biology-assets.anu.edu.au/GenAlEx/Welcome.html), and the level of genetic divergence between the dog and wolf subspecies was determined through the fixation index PhiPT for Y‐STRs alone and in combination with Y‐SNPs. *p*‐values were estimated after 9,999 permutations**.**

A combined analysis of Y chromosome SNP and STR markers was performed with the software NETWORK 5 (http://www.fluxus-engineering.com) using only the polymorphic loci. The Y‐STR loci were weighted inversely to their variance, and considering the much lower rate of substitutions relative to autosomal STR mutations; SNP loci were weighted as follows: transitions *w* = 10 and transversions *w* = 30. We first constructed a reduced‐median network and then applied a median‐joining analysis to create the final networks.

The haplotype data generated by the detection of 11 SNP loci in this study were also analyzed at a global scale with other worldwide *Canis* SNPs haplotypes described in Ding et al. (2012) and Brown et al. (2011). For the network analysis, we used the same method as described above.

#### 2.3.3 Mitochondrial sequences

The software Sequencher 4.8 (Gene Code Corporation, Ann Arbor, MI, USA) was used to edit and assemble the mtDNA sequence trace chromatograms. Sequence alignments were verified by eye and edited using MacClade 4.08 (http://macclade.org/index.html), and all sequences were collapsed to unique haplotypes using Fabox DNA Collapser (http://users-birc.au.dk/biopv/php/fabox/).

Analyses at the geographic scale of the Iberian Peninsula were performed considering 420 bp of the mtDNA fragment sequenced. Genetic diversity and differentiation analyses were performed with DnaSP v5.10.01 (http://www.ub.edu/dnasp/) (Librado & Rozas, 2009) for Iberian dogs and wolves for which mtDNA haplotype frequency information was available (135 dog sequences from a previous study (Pires et al., 2006) and Iberian wolves from this study). Dogs and wolves were considered as two populations and haplotype diversity (HD) and nucleotide diversity (*π*) were calculated for each population separately. The extent of divergence among these two populations and gene flow was also computed and statistically assessed. Because there are some indels in the sequence alignment, gaps were treated as a fifth character.

To test if differences in haplotype richness between wolves and dogs found in Iberia were due to unbalanced sampling, the total number of haplotypes for each population was estimated using a rarefaction resampling method as implemented in the software Analytic Rarefaction 2.0 (http://www.huntmountainsoftware.com/html/rarefaction.html).

To assess how Iberian wolves are related to other gray wolves and to dogs native to the Iberian Peninsula and neighboring areas, additional mtDNA control region I sequences of wolves, native dog breeds, and village dogs from Iberia and North Africa were retrieved from GenBank (GenBank accession numbers: AY706476–524, AF098115‐116, AF098123‐124, EF380226‐229, FJ978005‐8035, AF338808‐809, AF338812, AB007372 and Vilà et al., 1999 haplotypes lu1‐lu34). Sequences generated in this study that had no exact match to sequences deposited in public nucleotide databases were submitted to GenBank.

For worldwide comparisons, sequences were trimmed to 230 bp, a mtDNA control region fragment common to wolf sequences across many studies (*e.g.,* Pilot et al., 2010). Bayesian inference (BI) methods were used to reconstruct the phylogenetic relationships among the mtDNA sequences using *Canis latrans* as outgroup (GenBank accession no. DQ480510.1). The program jModelTest v.0.1.1 (http://computing.bio.cam.ac.uk/software.html) was used to select the model of evolution that best explains the nucleotide variation within the data set, excluding the outgroup and using the Akaike information criterion (AIC). The software MrBayes 3.1.2 (http://mrbayes.sourceforge.net/) was used to perform the BI analyses and two independent analyses, starting from different random trees, were run in parallel for 10^7^ generations, with four simultaneous Markov chains, sampling every 100th generation and discarding as burn‐in the first 25% of samples. As convergence diagnostics, we used the average standard deviation of split frequencies (<0.01) and inspected the plot of the log likelihood values (no increasing or decreasing tendency over time).

Additionally, the software NETWORK 5 was also used to investigate how the mtDNA haplotypes of Iberian *Canis*, the focus of this study, are related. A total of 46 haplotypes were considered for this analysis consisting of the same 420 bp data set as mentioned earlier for the BI analyses, with the following exceptions: no outgroup was used, and for sequences of different sizes that were the same for the nucleotides sequenced, only the longest one was considered. A median‐joining network was constructed applying a transversion/transition weight of 3:1 and default values for the remaining parameters; information concerning haplotype frequency was only used for the haplotypes sequenced in this study.

The absolute number of nucleotide differences among haplotypes was determined using the software PAUP version 4.0b10 (http://paup.csit.fsu.edu/).

## 3 Results

### 3.1 Autosomal STRs

Autosomal STRs analysis for dogs and wolves revealed a pairwise *F*_ST_ value of 0.112 (*p* = .000). The partitioning analysis using the Bayesian clustering procedure clearly shows a sharp separation between dogs, (village dogs and breed dogs, these latter including livestock guard dogs), and the Iberian wolf (Figure S1, orange and yellow, respectively). The average Q‐value (proportion of membership of each predefined population in each of the two clusters) for the studied populations is high for dogs (0.994 ± 0.0096) as well as for wolves (0.993 ± 0.0134). We did not detect any individual with an intermediate assignment value (threshold value considered was 95%).

### 3.2 Paternal lineages in Iberian *Canis*

Sequence data, for the studied loci for a subset of dogs (*n*_total_ = 59) and wolves (*n*_total_ = 44), did not reveal additional polymorphic sites (GenBank accession numbers GQ366706‐GQ366793 and KT967955‐KT967970). For female samples, no PCR products were obtained for any locus confirming the Y chromosome specificity of the designed primers.

Complete genotypes were obtained for 11 Y chromosome SNPs and four Y chromosome STRs for a total of 108 male canid samples, comprising 81 unrelated dogs (Iberian breed dogs, *n* = 62; African dogs, *n* = 11; Iberian village dogs, *n* = 8) and 27 Iberian wolves (Table S4A,B).

Replicate assays proved the reproducibility of the method, and SNaPshot methodology was applied successfully in two multiplex reactions.

**Figure 2:**
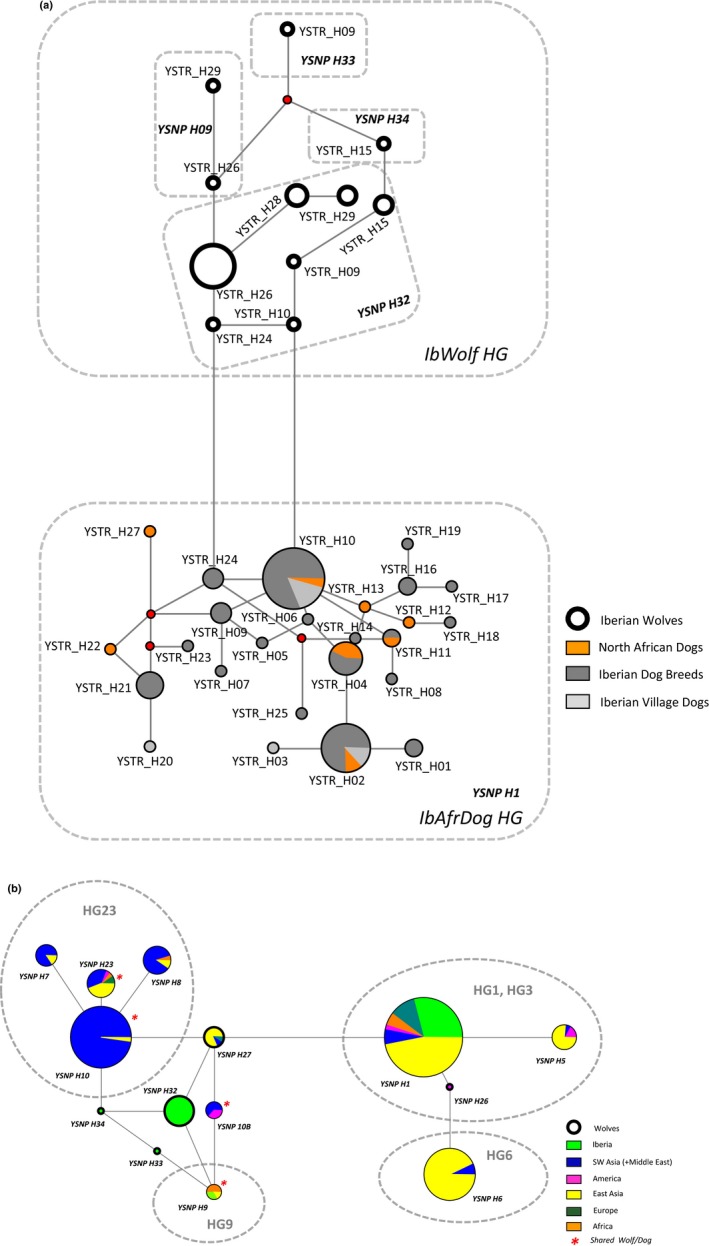
(**a**) Median‐joining Network displaying *Canis* Y chromosome haplotype diversity in Iberia based on six SNP and four STR loci. A total of 108 male individuals were analyzed comprising 81 domestic dogs (Iberian breed dogs *n* = 62, African dogs *n* = 11, Iberian village dogs *n* = 8) and 27 Iberian wolves. Thirty‐six haplotypes are displayed, 11 belonging to Iberian wolves and 25 to domestic dogs. Red dots represent theoretical median vectors introduced by the network software. SNP haplotype nomenclature (*YSNP H1*,* YSNP H9*,* YSNP H32*,* YSNP H33,* and *YSNP H34*) as in Ding et al. (2012). *IbWolf HG*—Iberian wolf haplogroup; *IbAfrDog HG*—Iberian and North African dog haplogroup. See Tables S4A,B and S5 for a list of Y‐SNP and Y‐STR haplotypes found per species and dog breed/population. (**b**) Median‐joining network displaying *Canis* Y chromosome haplotype worldwide diversity based on 11 Y‐SNPs. A total of 563 male individuals were considered (517 domestic dogs and 46 wolves) displaying 14 haplotypes (indicated in italics). Haplogroup nomenclature (HG1, HG3, HG6, HG9, and HG23) as in Ding et al. (2012). See Table S4A for a list of Y‐SNP haplotypes found worldwide

Of the 11 dog Y‐SNPs studied, only six were polymorphic within the Iberian and North African context: Ydog_21, Ydog_28A, Ydog_28B, Ydog_28C, Ydog_B, and Ydog_G1B loci (Table 2). Two major haplogroups were identified based on polymorphic Y chromosome SNPs (six loci) and STRs (four loci), one including Iberian and North African dogs (*IbAfrDog HG*) and another including Iberian wolves (*IbWolf HG*) (Figure 2a). These two haplogroups differ by four diagnostic mutations: an A(dog)/G(wolf) transition at locus Ydog_28A, an A/C transversion at locus Ydog_28B, a C/T transition at locus Ydog_B, and a C/A transversion at locus Ydog_G1B (Table 2).

Based on SNPs alone, the *IbAfrDog HG* comprises a single lineage (*YSNP H1*), whereas the *IbWolf HG* comprises four lineages: *YSNP H9*,* YSNP H32*,* YSNP H33,* and *YSNP H34*, following the nomenclature of Ding et al. (2012).

Regarding STRs alone, a total of 25 haplotypes are described for the studied dogs (Table S4B). The Iberian breed dogs share four haplotypes with North African dogs and two with Iberian village dogs. Exclusive haplotypes can be detected in each subgroup of dogs: 13 for Iberian breed dogs, four for North African dogs, and two for Iberian village dogs (Figure 2a, Table S4B). The haplotypic diversity (HD) (unbiased) ± *SE* is 0.59 ± 0.11 for African dogs, 0.52 ± 0.11 for Iberian breed dogs, 0.46 ± 0.08 for Iberian village dogs, and 0.52 ± 0.05 for the group of all studied domestic dogs. PhiPT pairwise values among these groupings of dogs varied between 0 (for Iberian breeds and village dogs) and 0.052 (for Iberian village dogs and African dogs). These values were not significant (*p*‐values > .05).

The Iberian wolf displays seven Y‐STR haplotypes, three of these are shared with dogs and four are exclusive to the Iberian wolf (Table S4B). The HD (unbiased) ± *SE* for the Iberian wolf is smaller compared to the dog population and is 0.41 ± 0.04. The PhiPT pairwise value between dogs and Iberian wolves of this study is 0.27 (*p* = .00).

The combination of 10 informative Y chromosome loci (four STRs plus six SNPs) allowed the identification of 36 haplotypes. Within the *IbAfrDog HG* and the *IbWolf HG*, 25 and 11 haplotypes were disclosed, respectively (Figure 2a; Table S4A,B). The HD (unbiased) ± *SE* values considering both Y markers were similar between dogs and Iberian wolves and were 0.14 ± 0.07 and 0.13 ± 0.05, respectively. The PhiPT pairwise value between dogs and Iberian wolves increased to 0.70 (*p* = .00).

The 11 Y chromosome SNPs from this study were also analyzed at a global geographic scale considering other worldwide *Canis* data available from Ding et al. (2012) and Brown et al. (2011) for a total of 517 dogs and 46 wolves. A total of 14 haplotypes were detected (Figure 2b, Table S4A). Dog haplotypes could be grouped into four haplogroups: HG1 & HG3 (are merged), HG6, HG9 and HG23 (haplogroup nomenclature as in Ding et al., 2012). Four haplotypes—*YSNP H9, YSNP H10, YSNP 10B and YSNP H23*—were shared among wolves (from Iberia, China, Iran, and Canada) and dogs (mostly sampled in southwest Asia and the Middle East) (Figure 2b; Table S4A). In particular, haplotype *YSNP H9* is shared between two Iberian wolves (samples IbWolf‐22 and IbWolf‐24), and dogs from Africa (Basenji dog breed, southern Africa) and East Asia (East Siberian Laika dog breed, East Asia). The *YSNP H9* haplotype differs from *YSNP 10B*, another haplotype shared between wolves and dogs, by a single SNP (locus B) (Table S4A).

The single Y‐SNP haplotype shared between Iberian and northwest African dogs (this study) is the most common haplotype among dogs worldwide—*YSNP H1* (Figure 2b; Table S4A), also found in a high proportion of East Asian dogs (47%).

### 3.3 Distinctiveness of Iberian wolf paternal lineages

We detected Y chromosome SNP haplotypes exclusive to the Iberian wolf. The three distinct Y chromosome SNP haplotypes of the Iberian wolves—*YSNP H32*,* H33,* and *H34*—segregate in the network among haplogroups HG1 & HG3, HG9, and HG23 where several unsampled nodes were previously detected by Brown et al. (2011) and Ding et al. (2012) (Figure 2b). Moreover, it is the first time that the haplogroup HG9, represented by the haplotype *YSNP* H9, is reported for wolves. Independent molecular markers, such as autosomal STRs and mtDNA, support that these HG9 samples were collected from wolf specimens.

### 3.4 Maternal lineages in Iberian *Canis*

**Figure 3:**
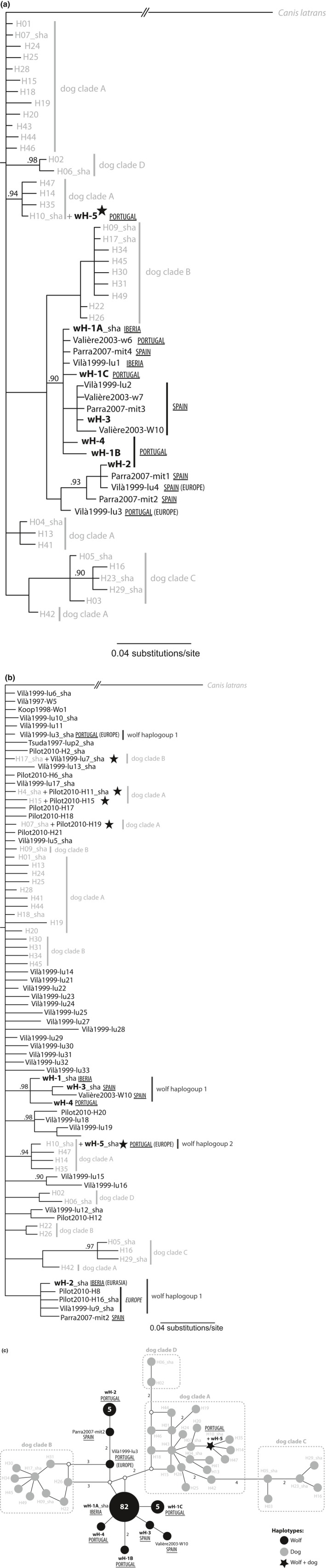
Phylogenetic relationships among wolf and dog mtDNA haplotypes. Bayesian Inference tree based on a 420 bp (**a**) and on a 230 bp mitochondrial fragment of the control region I (**b**) of Iberian and worldwide wolves, respectively. Dog haplotypes found in Iberia and North Africa were also included (Pires et al., 2006). Posterior probability support values ≥ 0.90 are shown. Outgroup and dog haplotypes are in gray; wolf haplotypes are in black. When originally distinct haplotypes within a species were collapsed to the same 420 bp or 230 bp haplotype, the label includes a representative haplotype followed by “sha” (shared). Wolf haplotype labels, for sequences retrieved from GenBank, consist of author name and date followed by original haplotype code (see text for accession number). Iberian wolf sequences generated in this study are labeled wH‐1 to wH‐5 (bold); haplotypes that collapsed to the same haplotype when trimmed to 230 bp have the same root label followed by a letter (*e.g*., wH‐1A, wH‐1B and wH‐1C are indistinct when trimmed to 230 bp). Dog haplotype nomenclature (H01‐H49) as in Pires et al. 2006; dog clades (a–d) as in Savolainen et al. 2002. Geographic distribution within the Iberian Peninsula (Portugal, Spain, or Iberia) and overall geographic distribution (in parenthesis) is indicated for wolf haplotypes; for the worldwide BI tree (b) geographic distribution is also shown for wolf haplotypes that group with Iberian wolves (in italics); wolf haplogroup nomenclature (1 and 2) as in Pilot et al., 2010 is shown for Iberian wolves haplotypes and other wolf haplotypes that group with them. 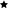 ‐ haplotype shared between wolf and dog. (**c**) Median‐joining network of a 420 bp mitochondrial fragment of the control region I of Iberian wolves (subset of the data used in Figure 3 A—see text for details). Dog and wolf haplotype labels as in Figures 3 a and b. Frequency of haplotypes is only shown for wolves sequenced in this study—circle size proportional to frequency (% of occurrence inside the circle); remaining haplotypes represent sequences downloaded from GenBank (no frequency information available). Small white circles represent missing haplotypes (median vectors), and link length between nodes is proportional to nucleotide differences (number of mutations is shown next to the link when >1)

The 56 Iberian wolf samples revealed seven haplotypes for a 420 bp fragment of the mtDNA control region I: wH‐1A (frequency 82.1%), wH‐1B (1.8%), wH‐1C (5.4%), wH‐2 (5.3%), wH‐3 (1.8%), wH‐4 (1.8%), and wH‐5 (1.8%), where wH‐1A is the most frequent haplotype found throughout Portugal and Spain (Table 3, Figures 3a,c; Table S5).

**Table 3:** Summarized description of mitochondrial DNA haplotypes based on 420 bp of control region I identified in the 56 Iberian wolves sequenced. For detailed information per individual sample, see Table S5

Haplotype code^a^	Haplotype geographic distribution	Haplotype frequency (%)
wH‐1A	Portugal (Aveiro, Braga, Bragança, Guarda, V.Castelo, V.Real, Viseu); Spain (X.Lima, Zamora)	82.1
wH‐1B	Portugal (V.Castelo)	1.8
wH‐1C	Portugal (Bragança, Viseu)	5.4
wH‐2	Portugal (Bragança, V.Real)	5.3
wH‐3	Spain (Galicia)	1.8
wH‐4	Portugal (V.Real)	1.8
wH‐5	Portugal (Braga)	1.8

a For the wolf samples sequenced, longer haplotypes (420 bp) that collapsed to the same haplotype when trimmed to 230 bp were given the same root label followed by a letter, for example, wH‐1A, wH‐1B, and wH‐1C are indistinct when trimmed to 230 bp.

Considering the larger sequence fragments available for Iberian wolves, our findings increase the total number of mtDNA control region I haplotypes reported for Iberian wolf to 24 (see Vilà et al., 1997, 1999; Randi et al., 2000; Valière et al., 2003; Björnerfeldt, Webster, & Vilà, 2006, unpublished GenBank accession numbers EF380226‐9, and Godinho & Ferrand, 2007). Of the 24 haplotypes described for Iberia, two are found in both Portugal and Spain, nine are found in Portugal (five of them exclusive to Portugal), and 13 are found in Spain (11 of them exclusive to Spain).

Five of the seven 420 bp mtDNA haplotypes found in this study were identified for the first time in Iberian wolves (wolf haplotypes wH‐1B, wH‐1C, wH‐2, wH‐3 and wH‐4 in this study) and were submitted to GenBank (accession numbers JX845621‐JX845625, where JX845621 may correspond to Godinho & Ferrand, 2007 haplotype D); one had already been identified in Spain (this study haplotype wH‐1A; Björnerfeldt et al., 2006 haplotype isolate 1); and one is shared with dogs (Portuguese native dog breeds—Castro Laboreiro Watchdog, Portuguese Warren hound, Alentejo mastiff, and Portuguese Sheepdog; Spanish dogs—Spanish mastiff; and dogs from Tunisia) (this study wolf haplotype wH‐5 from sample IbWolf‐05; dog haplotypes H10 and H48, dog clade A in Pires et al., 2006) (Figures 3a,c; Table S5).

Wolf sequences generated in this study diverged by 1 to 9 nucleotides compared to other wolf sequences reported for the Iberian Peninsula, the most divergent haplotype being the one that is shared with dogs (this study wolf haplotype wH‐5, Pires et al., 2006 dog haplotypes H10 and H48).

For the overlapping 420 bp fragment in 164 dog samples from Iberia and North Africa sequenced in a previous study (Pires et al., 2006; original size fragment 887 bp), we found 37 haplotypes (Figures 3a,c; Table S5). When the shorter overlapping fragment of 230 bp was considered for a wider geographical range comparison (wolves worldwide), we found eight haplotypes for Iberian wolves and 27 haplotypes for dogs (Figure 3b; Table S5). Within the eight 230 bp haplotypes identified across studies for Iberian wolves, five of them were still unique to the Iberian Peninsula (this study wolf haplotypes wH1, wH3 and wH4; haplotype MIT2—GenBank accession number EF380216; and haplotype w10—Valière et al., 2003), three were restricted to Spain (this study haplotype wH3; haplotype w10—Valière et al., *2003*; and haplotype MIT2), and one was only found in Portugal (this study haplotype wH4) (Figure 3b). All the wolf samples sequenced in this study, when trimmed to 230 bp, segregate within the wolf clade previously reported for Iberia (Pilot et al., 2010), except for one individual (this study haplotype wH‐5) (Figure 3b). This exception corresponds to the only mtDNA haplotype that is shared between Iberian wolves and dogs, representing one of the five cases of wolf/dog haplotype sharing detected in our worldwide wolf analysis (Figure 3b). The same occurs for the 420 bp fragment (this study wolf haplotype wH‐5; and dog haplotypes H10 and H48 from Pires et al., 2006) (Figure 3a).

In both phylogenetic analyses, either considering the 420 bp or the 230 bp fragment (model of evolution GTR+I+G and TPM1uf+I+G, respectively), the Iberian wolf sequences generated in this study cluster with other wolf sequences forming clades with high node support (posterior probability ≥0.90), except for: haplotype wH‐2 in the 230 bp worldwide analysis that forms a group with low support with other wolf sequences; and haplotype wH‐5 (shared with dogs) that forms a group with high node support with other dog sequences (dog haplogroup A) (posterior probability 0.94) (Figures 3a,b).

For the 230 bp fragment, the wolf samples sequenced in this study diverged by 1 to 10 nucleotides compared to other wolf sequences worldwide, and the most divergent Iberian wolf haplotype sequence was also found in other European and Asian wolves (this study wolf haplotype wH‐2 and haplotype W3—Pilot et al., 2010).

Concerning dogs and wolves found in Iberia for which we have haplotype frequency information (135 dogs, 56 wolves), the rarefaction analysis revealed that haplotype richness was approximately 2.5 times higher for dogs compared to the wolves (18.8 vs. 7). Haplotype diversity (HD) for the Iberian dogs was also approximately three times higher than the value computed for Iberian wolves, 0.92 ± 0.008 (mean ± *SD*) and 0.32 ± 0.080, respectively. The difference in nucleotide diversity (*π*) for dogs and wolves found in Iberia is even higher, 0.014 ± 0.0006 (mean ± *SD*) and 0.002 ± 0.0008, respectively.

The statistical test indicates that Iberian dogs and wolves are strongly genetically differentiated (χ^2^ = 186.428, *p* = .0000), where the *F*_ST_ value between the two subspecies is 0.486.

## 4 Discussion

### 4.1 Genetic diversity of Iberian *Canis*

Maternal and paternal markers used in this study effectively reveal unreported diversity in extant dogs and wolves that inhabit Iberia and it reinforces the significant genetic differentiation between Iberian wolves and domestic dogs.

The number of Y‐STR haplotypes is higher than previously reported for male domestic dogs and Iberian wolves in south European *Canis* populations by Parra et al. (2008), Iacolina et al. (2010) and Godinho et al. (2011). HD values are similar between Iberian and Italian wolves (0.474 ± 0.056, Iacolina et al., 2010). HD values for Portuguese dogs are within the range reported by Bannasch et al. (2005) for a sample of 824 males including breed dogs and mongrels (average HD for breeds 0.38 ± 0.03 and for mongrels 0.95 ± 0.03).

The Y chromosome data provide an update to the most parsimonious phylogenetic tree presented by Ding et al. (2012) with the addition of new distinct haplotypes for Iberian wolves (*Canis lupus signatus*).

The studied Iberian dogs belong to HG1, one of the haplogroups universally shared by dogs (as in Ding et al., 2012), suggesting a common origin with other European dogs. Moreover, HG1 is also present in the northwestern African dog population analyzed, denoting the same patrilineal origin as Iberian dogs. Our results are in agreement with the findings of Ding et al. (2012) for North African dog breeds but contrast with the results of Brown et al. (2011) for African dog populations from more southern regions (Africans/South Africa, Basenji/Central Africa and Rhodesian ridgeback/southern Africa).

Although an extensive study on several gray wolf populations is yet to be performed for Y chromosome markers, the Iberian wolf subspecies exhibits high diversity with three distinct Y chromosome SNP lineages not found in any other wolves or dogs sampled so far. Therefore, the contemporary Iberian wolf subspecies retains significant Y chromosome diversity.

Iberian dogs revealed one‐fourth of the Y‐SNP lineages detected in Iberian wolves. This result may be related to ancient events such as domestication, as a very low mutation rate is estimated for the Y chromosome among mammal species (Kumar & Subramanian, 2002). For dogs, a mutation rate of 1.65 × 10^−9^ substitutions per year and base pair, similar to humans, was assumed (Natanaelsson et al., 2006). The higher level of variability of Y‐STRs observed in dogs is associated with the nature of the marker (higher mutational rate than for SNPs) and with the demographic expansion observed for this subspecies compared to the Iberian wolf's bottleneck in recent times (Sastre et al., 2011).

Two Iberian wolf samples share a low frequency patrilineage with dogs from South Africa and East Asia, the haplogroup HG9. This finding supports a wolf origin for this dog clade firstly described by Natanaelsson and collaborators (Natanaelsson et al., 2006). It is probable that an extensive study of wolf specimens with Y chromosome markers will reveal other wolf populations carrying haplotypes segregating within the HG9 haplogroup. The possibility that these specimens are male dog x female wolf hybrids (the most probable direction of wolf–dog hybridization (Vilà et al., 2003; Godinho et al., 2011, 2015; but see also Hindrikson et al., 2012) is not supported by this study, as our survey on 74 male dogs in Iberia, including village dogs, revealed that they all belong to haplogroup HG1 with no exception, which is five SNPs apart from haplogroup HG9. Therefore, no Iberian dog analyzed so far carried Y chromosome haplotypes belonging to HG9. Moreover, these two particular wolves dated to 2008 carry typical wolf mtDNA haplotypes (wH‐1A and wH‐1B in this study), and typical wolf genotypes regarding autosomal microsatellite data. A sharp distinction between Iberian wolves and dogs was found using our dataset, which is in accordance with previous studies reporting levels of pairwise *F*_ST_ ranging from 0.193 to 0.341 (*p* < .0001) depending on the set of autosomal markers used (Godinho et al., 2011). Our autosomal molecular data also suggest a low level of recent admixture between both subspecies in Iberia, although historical gene flow cannot be rejected (Fan et al., 2016). Population partitioning using a Bayesian clustering procedure and based on microsatellites loci reveals complete segregation between Portuguese village dogs or livestock guard dogs and Iberian wolves genotypes (Figure S1), in contrast to the findings of Kopaliani and collaborators (Kopaliani et al., 2014) for the Caucasus region. It is important to emphasize here that our sampling strategy constrained the sampling of potential hybrids as we did not sample free‐ranging or feral dogs. Although wolf–dog hybrids are known to occur in the wild (Randi & Lucchini, 2002; Vilà & Wayne, 1999; Vilà et al., 2003), including in Iberia (Godinho et al., 2011, 2015 but see also Echegaray & Vilà, 2010), hybridization is still a limited phenomenon in Iberia (Fan et al., 2016; Godinho et al., 2011, 2015). Moreover, the search for the melanistic *K* locus mutation, a three nucleotide deletion derived from dogs (Caniglia et al., 2013; Randi et al., 2014), in 42 wolf tissue samples from this study and 26 forensic swabs (swabbed livestock wounds from wolf attacks) collected between 1991–2015 north and south of the Douro river in Portugal, revealed no animals with a hybrid genotype (Quaresma, 2016).

The seven mtDNA haplotypes described in this study, five of them unique to Iberian wolves, add to the diversity previously reported for this small and isolated gray wolf population. The fact that the mtDNA sequences trimmed to 230 bp still revealed two new haplotypes for Iberian wolves found in Portugal expands diversity previously reported for Iberia within the wolf haplogroup 1 (Pilot et al., 2010) and adds representation of wolf haplogroup 2 (Pilot et al., 2010). This reinforces the evidence for unreported diversity in Iberian wolves. Low genetic diversity has been reported for wolf populations that have experienced population decline and fragmentation (Randi et al., 2000). Wolf populations in Italy, Scandinavia, Switzerland, and France all have fewer haplotypes than the Iberian population (Ellegren, Savolainen, & Rosen, 1996; Pilot et al., 2010; Randi et al., 2000). This can most likely be explained by the fact that, although the Iberian wolf population, as other European wolf population, has been experiencing demographic decline since the Late Pleistocene (Pilot et al., 2014) and underwent a steep human‐mediated decline in the early 20th century, its effective population size was never as low as in other regions.

### 4.2 Genetic differentiation of *Canis* subspecies

Y chromosome molecular markers disclose a genetic distinctiveness of the Iberian wolf from other wolves and from domestic dogs. Four Y‐SNP markers are diagnostic and can segregate Iberian wolves from male dog samples. This result obtained from contemporaneous samples does not support a local domestication event for dogs in Iberia, as the genetic makeup of local dogs is apparently made of a myriad of Y chromosome lineages (including Y‐STRs) that came from elsewhere. In any case, breeds have a very recent origin when compared to the domestication of dogs, and the haplotypes that are currently present in native breeds may not be representative of the haplotypes that were present in early dog subspecies. Further investigation using historical and ancient samples and high‐resolution markers (*e.g*., complete mtDNA genomes, genomewide SNPs), is necessary for the reconstruction of the history of *Canis* subspecies in Iberia and for investigating associated population evolutionary processes.

The mentioned four diagnostic Y‐SNP markers can easily be used to screen noninvasive samples such as scats, hairs, and forensic swabs for their species origin. The resulting information is crucial for monitoring the wild Iberian wolf metapopulation. Moreover, these SNPs can be incorporated in a single panel of markers to be developed for the harmonization of molecular markers useful in studying the population genetics of *Canis lupus* (de Groot et al., 2016).

The genetic differentiation of Iberian wolves found for Y chromosome markers is probably due to diverse geographic and genetic factors, such as their isolation from other European wolf populations and consequent genetic drift. As mentioned earlier, some wolf diversity may have been lost during the severe population decline that occurred in the 20th century, and therefore, we hypothesize that the ancestral diversity of the Iberian wolf patrilineal lineages could have been higher.

We report mtDNA haplotype sharing between wolves and dogs worldwide and in the Iberian Peninsula. In Iberia, the shared haplotype wH‐5 segregates within the most common dog mtDNA clade A found in dogs from Portugal. Furthermore, the closest mitochondrial haplotypes to the Iberian wolf haplotype wH‐5 are other dog haplotypes differing by a single transition mutation.

The identification of a shared mtDNA haplotype between Iberian wolf and dog breeds may be interpreted as introgression of dog haplotypes into the Iberian wolf genetic pool. This particular sample (IbWolf‐5, male) was collected in the year 2000 within the known wolf range in the north of Portugal (Serra da Cabreira), from a deceased animal with a typical wolf phenotype, showing no external signs of admixture with domestic dog. Probably due to poor sample preservation and thus DNA degradation, we were not able to generate microsatellite data for this sample, neither autosomal nor Y‐linked microsatellites. Nevertheless, this sample exhibits a wolf Y‐SNP profile: wolf‐typical nucleotides were detected at the described diagnostic loci. Being a male wolf, for this sample to belong to a first‐generation hybrid, it could have only resulted from the cross between a female dog and a male wolf, which is considered rare. The first record of this kind of hybrid in Europe dates from 2008–2009 in Latvia, a single specimen, which was reported in Hindrikson et al. (2012). Given the high genetic distance from all other Iberian wolf mtDNA haplotypes and the fact that it has never been previously found in any other wolf population, an older hybridization event cannot be excluded. Alternatively, the shared haplotype may represent an ancestral variant preserved until recently, as this haplotype segregates within wolf haplogroup 2, which was extensively detected in ancient wolf samples from western Europe (Pilot et al., 2010). Once again, the study of historical and ancient samples from the Iberian Peninsula will help to clarify this issue and possibly to date the oldest evidence of this haplotype in Iberian wolves.

### 4.3 Impacts for the conservation of the Iberian wolf

We have shown, using both nuclear and mitochondrial data, that the Iberian wolf displays greater diversity than previously reported, with new maternal and paternal haplotypes disclosed.

We hypothesize that mtDNA haplotypes shared between wolves and dogs may represent relic common ancestral *Canis* lineages that did not diverge after the domestication of dogs. Although hybridization has been recorded in Iberia, the fact that in our dataset the percentage of dog–wolf mtDNA haplotype sharing in this area is roughly half of what was found elsewhere supports the conclusion that hybridization in Iberia, more specifically in Portugal (as most wolf samples are from this region), is a rare event. If disturbance of wolf habitats in Iberia continues, due to both habitat loss and/or fragmentation, in addition to an increase in the feral dog population, which largely overlaps with the wolf range (Álvares, 2011; LIFE Co‐EX 2008), the genetic identity of the Iberian wolf is threatened.

Wolves found in Iberia represent a reservoir of unique genetic and ecomorphologic variants of the gray wolf. Therefore, according to Hofreiter and Barnes' (2010) proposal, this stresses the need for maintenance and even intensification of multiple measures toward the long‐term sustainability of this locally adapted, isolated, and peripheral population, the Iberian wolf.

## Conflict of Interest

None declared.

## Data Archiving

Mitochondrial DNA sequences: GenBank accession numbers JX845621‐JX845625. Y chromosome DNA sequences: GenBank accession numbers GQ366706‐GQ366793 and KT967955‐KT967970.

## References

[ece32949-bib-0001] Álvares, F. J. (2011). Ecologia e conservação do lobo (Canis lupus, L.) no Noroeste de Portugal. PhD thesis. Lisbon University. Portugal.

[ece32949-bib-0003] van Asch, B. , et al. (2005). MtDNA diversity among four Portuguese autochthonous dog breeds: A fine‐scale characterisation. BMC Genetics, 6(1), 37. 15972107 10.1186/1471-2156-6-37 PMC1180824

[ece32949-bib-0004] van Asch, B. , et al. (2010). Genetic profiles and sex identification of found‐dead wolves determined by the use of an 11‐loci PCR multiplex. Forensic science international. Genetics, 4(2), 68–72. 10.1016/j.fsigen.2009.05.003 20129463

[ece32949-bib-0005] Bannasch, D. L. , et al. (2005). Y chromosome haplotype analysis in purebred dogs. Mammalian Genome, 16(4), 273–280. 15965788 10.1007/s00335-004-2435-8

[ece32949-bib-0006] Björnerfeldt, S. , Webster, M. , & Vilà, C. (2006). Relaxation of selective constraint on dog mitochondrial DNA following domestication. Genome Research, 16(8), 990–994. 16809672 10.1101/gr.5117706 PMC1524871

[ece32949-bib-0010] Brown, S. K. , et al. (2011). Phylogenetic distinctiveness of middle eastern and southeast Asian village dog Y chromosomes illuminates dog origins B. Fenton, ed. PLoS ONE, 6(12), e28496. 22194840 10.1371/journal.pone.0028496 PMC3237445

[ece32949-bib-0011] Cabrera, A. (1907). Los lobos de España. Boletín de la Real Sociedad Española de Historia Natural, 7, 193–197.

[ece32949-bib-0012] Caniglia, R. , et al. (2013). Black coats in an admixed wolf × dog pack is melanism an indicator of hybridization in wolves? European Journal of Wildlife Research, 59(4), 543–555.

[ece32949-bib-0013] Chapron, G. , et al. (2014). Recovery of large carnivores in Europe's modern human‐dominated landscapes. Science, 346(6216), 1517–1519. 25525247 10.1126/science.1257553

[ece32949-bib-0014] Detry, C. , & Cardoso, J. L. (2010). On some remains of dog (*Canis familiaris*) from the Mesolithic shell‐middens of Muge, Portugal. Journal of Archaeological Science, 37(11), 2762–2774.

[ece32949-bib-0015] Ding, Z.‐L. , et al. (2012). Origins of domestic dog in southern East Asia is supported by analysis of Y‐chromosome DNA. Heredity, 108(5), 507–514. 22108628 10.1038/hdy.2011.114 PMC3330686

[ece32949-bib-0016] Echegaray, J. , & Vilà, C. (2010). Noninvasive monitoring of wolves at the edge of their distribution and the cost of their conservation. Animal Conservation, 13(2), 157–161.

[ece32949-bib-0017] Eichmann, C. , Berger, B. , & Parson, W. (2006). Relevant aspects for forensic STR analysis of canine DNA: Repeat‐based nomenclature and sensitive PCR multiplexes. International Congress Series, 1288, 813–815.

[ece32949-bib-0018] Ellegren, H. , Savolainen, P. , & Rosen, B. (1996). The genetical history of an isolated population of the endangered grey wolf *Canis lupus*: A study of nuclear and mitochondrial polymorphisms. Philosophical Transactions: Biological Sciences, 351(1348), 1661–1669. 9004318 10.1098/rstb.1996.0148

[ece32949-bib-0019] Falush, D. , Stephens, M. , & Pritchard, J. K. (2003). Inference of population structure using multilocus genotype data: Linked loci and correlated allele frequencies. Genetics, 164(4), 1567–1587. 12930761 10.1093/genetics/164.4.1567 PMC1462648

[ece32949-bib-0020] Fan, Z. , et al. (2016). Worldwide patterns of genomic variation and admixture in gray wolves. Genome Research, 26(2), 163–173. 26680994 10.1101/gr.197517.115 PMC4728369

[ece32949-bib-0021] Francisco, L. V. , et al. (1996). A class of highly polymorphic tetranucleotide repeats for canine genetic mapping. Mammalian Genome, 7(5), 359–362. 8661717 10.1007/s003359900104

[ece32949-bib-0022] Ginja, C. , Telo da Gama, L. , & Penedo, M. C. T. (2009). Y chromosome haplotype analysis in Portuguese cattle breeds using SNPs and STRs. The Journal of Heredity, 100(2), 148–157. 18832111 10.1093/jhered/esn080

[ece32949-bib-0023] Ginja, C. , et al. (2010). Origins and genetic diversity of New World Creole cattle: Inferences from mitochondrial and Y chromosome polymorphisms. Animal Genetics, 41(2), 128–141. 19817725 10.1111/j.1365-2052.2009.01976.x

[ece32949-bib-0025] Godinho, R. , & Ferrand, N. (2007). Estudo da diversidade e estruturação genética das populações de lobos (Canis lupus) em Portugal. Technical Report, Porto, Portugal.

[ece32949-bib-0026] Godinho, R. , et al. (2011). Genetic evidence for multiple events of hybridization between wolves and domestic dogs in the Iberian Peninsula. Molecular Ecology, 20(24), 5154–5166. 22066758 10.1111/j.1365-294X.2011.05345.x

[ece32949-bib-0027] Godinho, R. , et al. (2015). Real‐time assessment of hybridization between wolves and dogs: Combining non‐invasive samples with ancestry informative markers. Molecular Ecology Resources, 15(2), 317–328. 25132482 10.1111/1755-0998.12313

[ece32949-bib-0028] Gomerčić, T. , Sindičić, M. , & Florijančić, T. (2013). Differentiating between Y chromosome sequences in Croatian canids. Veterinary Archives, 83(5), 571–579.

[ece32949-bib-0029] Götherström, A. , et al. (2005). Cattle domestication in the Near East was followed by hybridization with aurochs bulls in Europe. Proceedings. Biological sciences/The Royal Society, 272(1579), 2345–2350. 10.1098/rspb.2005.3243 PMC1559968 16243693

[ece32949-bib-0030] de Groot, G. A. , et al. (2016). Decades of population genetic research reveal the need for harmonization of molecular markers: The grey wolf *Canis lupus* as a case study. Mammal Review, 46(1), 44–59.

[ece32949-bib-0031] Guyon, R. , et al. (2003). A 1‐Mb resolution radiation hybrid map of the canine genome. PNAS, 100(9), 5296–5301. 12700351 10.1073/pnas.0831002100 PMC154339

[ece32949-bib-0032] Hailer, F. , & Leonard, J. (2008). Hybridization among three native North American *Canis* species in a region of natural sympatry. PLoS ONE, 3(10), e3333. 18841199 10.1371/journal.pone.0003333 PMC2556088

[ece32949-bib-0034] Hellborg, L. (2004). Evolutionary Studies of the Mammalian Y Chromosome. PhD thesis, Uppsala University, Sweden. Acta Universitatis Upsaliensis.

[ece32949-bib-0035] Hindrikson, M. , et al. (2012). Bucking the trend in wolf‐dog hybridization: First evidence from europe of hybridization between female dogs and male wolves. PLoS ONE, 7(10), e46465. 23056315 10.1371/journal.pone.0046465 PMC3463576

[ece32949-bib-0036] Hofreiter, M. , & Barnes, I. (2010). Diversity lost: Are all Holarctic large mammal species just relict populations? BMC Biology, 8(1), 46. 20409351 10.1186/1741-7007-8-46 PMC2858106

[ece32949-bib-0037] Holmes, N. G. , et al. (2009). Eighteen canine microsatellites. Animal Genetics, 26(2), 132a–133. 10.1111/j.1365-2052.1995.tb02659.x 7733507

[ece32949-bib-0038] Iacolina, L. , et al. (2010). Y‐chromosome microsatellite variation in Italian wolves: A contribution to the study of wolf‐dog hybridization patterns. Mammalian Biology, 75(4), 341–347.

[ece32949-bib-0039] Kopaliani, N. , et al. (2014). Gene flow between wolf and shepherd dog populations in Georgia (Caucasus). The Journal of Heredity, 105(3), 345–353. 24622972 10.1093/jhered/esu014

[ece32949-bib-0040] Kumar, S. , & Subramanian, S. (2002). Mutation rates in mammalian genomes. Proceedings of the National Academy of Sciences of the United States of America, 99, 803–808. 11792858 10.1073/pnas.022629899 PMC117386

[ece32949-bib-0041] Lescureux, N. , & Linnell, J. D. C. (2014). Warring brothers: The complex interactions between wolves (*Canis lupus*) and dogs (*Canis familiaris*) in a conservation context. Biological Conservation, 171, 232–245.

[ece32949-bib-0042] Librado, P. , & Rozas, J. (2009). DnaSP v5: A software for comprehensive analysis of DNA polymorphism data. Bioinformatics, 25(11), 1451–1452. 19346325 10.1093/bioinformatics/btp187

[ece32949-bib-0043] LIFE Co‐EX (2008). IMPROVING COEXISTENCE OF LARGE CARNIVORES AND AGRICULTURE IN S‐EUROPE. Report of Activities Annex 4, Action A9 ‐ Assessment of the presence and distribution of stray dogs. Retrieved from http://www.life-coex.net/Final-Technical-Report/Annex 14 ‐ Action D4.pdf

[ece32949-bib-0044] López‐Bao, J. V. , et al. (2015). Toothless wildlife protection laws. Biodiversity and Conservation, 24(8), 2105–2108.

[ece32949-bib-0045] Meadows, J. R. S. , et al. (2006). Globally dispersed Y chromosomal haplotypes in wild and domestic sheep. Animal Genetics, 37(5), 444–453. 16978172 10.1111/j.1365-2052.2006.01496.x

[ece32949-bib-0046] Mellersh, C. S. , et al. (1997). A linkage map of the canine genome. Genomics, 46(3), 326–336. 9441735 10.1006/geno.1997.5098

[ece32949-bib-0047] Montgomery, G. W. , & Sise, J. A. (1990). Extraction of DNA from sheep white blood cells. New Zealand Journal of Agricultural Research, 33(3), 437–441.

[ece32949-bib-0048] Moore, M. , & Sacks, B. N. (2010). Thirty‐one short red fox (*Vulpes vulpes*) microsatellite markers. Molecular Ecology Resources, 10, 404–408.21565039

[ece32949-bib-0049] Natanaelsson, C. , et al. (2006). Dog Y chromosomal DNA sequence: Identification, sequencing and SNP discovery. BMC Genetics, 7(1), 45. 17026745 10.1186/1471-2156-7-45 PMC1630699

[ece32949-bib-0050] Neff, M. W. , et al. (1999). A second‐generation genetic linkage map of the domestic dog, Canis familiaris. Genetics, 151(2), 803–820. 9927471 10.1093/genetics/151.2.803 PMC1460484

[ece32949-bib-0051] Pang, J.‐F. , et al. (2009). mtDNA data indicate a single origin for dogs south of Yangtze River, less than 16,300 years ago, from numerous wolves. Molecular Biology and Evolution, 26(12), 2849–2864. 19723671 10.1093/molbev/msp195 PMC2775109

[ece32949-bib-0052] Parra, D. , et al. (2008). Genetic differentiation in pointing dog breeds inferred from microsatellites and mitochondrial DNA sequence. Animal Genetics, 39(1), 1–7. 18254732 10.1111/j.1365-2052.2007.01658.x

[ece32949-bib-0053] Peakall, R. , & Smouse, P. E. (2006). Genalex 6: Genetic analysis in Excel. Population genetic software for teaching and research. Molecular Ecology Notes, 6(1), 288–295. 10.1093/bioinformatics/bts460 PMC3463245 22820204

[ece32949-bib-0054] Petit, E. , Balloux, F. , & Excoffier, L. (2002). Mammalian population genetics: Why not Y? Trends in Ecology & Evolution, 17(1), 28–33.

[ece32949-bib-0056] Pilot, M. , et al. (2010). Phylogeographic history of grey wolves in Europe. BMC Evolutionary Biology, 10(1), 104. 20409299 10.1186/1471-2148-10-104 PMC2873414

[ece32949-bib-0057] Pilot, M. , et al. (2014). Genome‐wide signatures of population bottlenecks and diversifying selection in European wolves. Heredity, 112(4), 428–442. 24346500 10.1038/hdy.2013.122 PMC3966127

[ece32949-bib-0058] Pimenta, V. et al. (2005). Situação populacional do lobo em Portugal: resultados do Censo Nacional 2002/2003 Technical Report, Lisbon, Portugal.

[ece32949-bib-0059] Pionnier‐Capitan, M. , et al. (2011). New evidence for Upper Palaeolithic small domestic dogs in South‐Western Europe. Journal of Archaeological Science, 38(9), 2123–2140.

[ece32949-bib-0060] Pires, A. , et al. (2006). Mitochondrial DNA sequence variation in Portuguese native dog breeds: Diversity and phylogenetic affinities. The Journal of Heredity, 97(4), 318–330. 16818467 10.1093/jhered/esl006

[ece32949-bib-0061] Pires, A. E. , et al. (2009). Molecular structure in peripheral dog breeds: Portuguese native breeds as a case study. Animal Genetics, 40(4), 383–392. 19298456 10.1111/j.1365-2052.2009.01849.x

[ece32949-bib-0062] Pritchard, J. K. , Stephens, M. , & Donnelly, P. (2000). Inference of population structure using multilocus genotype data. Genetics, 155(2), 945–959. 10835412 10.1093/genetics/155.2.945 PMC1461096

[ece32949-bib-0063] Quaresma, A. (2016). Non‐invasive molecular genetzrics studies of Iberian wolves in Alvão/Padrela (Master thesis). Lisbon University, Portugal.

[ece32949-bib-0064] Ramasamy, R. , et al. (2014). STRUCTURE PLOT: A program for drawing elegant STRUCTURE bar plots in user friendly interface. SpringerPlus, 3(1), 431. 25152854 10.1186/2193-1801-3-431 PMC4141070

[ece32949-bib-0065] Ramirez, O. , et al. (2006). Genetic assessment of the Iberian wolf *Canis lupus* signatus captive breeding program. Conservation Genetics, 7(6), 861–878.

[ece32949-bib-0067] Randi, E. , & Lucchini, V. (2002). Detecting rare introgression of domestic dog genes into wild wolf (*Canis lupus*) populations by Bayesian admixture analyses of microsatellite variation. Conservation Genetics, 3(1), 29–43.

[ece32949-bib-0068] Randi, E. , et al. (2000). Mitochondrial DNA variability in Italian and East European wolves: Detecting the consequences of small population size and hybridization. Conservation Biology, 14(2), 464–473.

[ece32949-bib-0069] Randi, E. , et al. (2014). Multilocus detection of wolf x dog hybridization in italy, and guidelines for marker selection. PLoS ONE, 9(1), e86409. 24466077 10.1371/journal.pone.0086409 PMC3899229

[ece32949-bib-0071] Richman, M. , et al. (2001). Characterization of a minimal screening set of 172 microsatellite markers for genome‐wide screens of the canine genome. Journal of Biochemical and Biophysical Methods, 47(1–2), 137–149. 11179770 10.1016/s0165-022x(00)00160-3

[ece32949-bib-0072] Sacks, B. N. , et al. (2013). Y chromosome analysis of dingoes and southeast asian village dogs suggests a neolithic continental expansion from southeast asia followed by multiple austronesian dispersals. Molecular Biology and Evolution, 30(5), 1103–1118. 23408799 10.1093/molbev/mst027

[ece32949-bib-0073] Sambrook, J. , Fritsch, E. , & Maniatis, T. (1989). Molecular cloning: A laboratory manual, 2nd ed New York, NY: Cold Spring Harbor Laboratory Press.

[ece32949-bib-0074] Sastre, N. , et al. (2011). Signatures of demographic bottlenecks in European wolf populations. Conservation Genetics, 12(3), 701–712.

[ece32949-bib-0075] Savolainen, P. , et al. (2002). Genetic evidence for an East Asian origin of domestic dogs. Science (New York, N.Y.), 298(5598), 1610–1613. 10.1126/science.1073906 12446907

[ece32949-bib-0076] Schregel, J. , et al. (2015). Y chromosome haplotype distribution of brown bears (*Ursus arctos*) in Northern Europe provides insight into population history and recovery. Molecular Ecology, 24(24), 6041–6060. 26769404 10.1111/mec.13448

[ece32949-bib-0077] Shibuya, H. , et al. (1994). A polymorphic (AGGAAT)n tandem repeat in an intron of the canine von Willebrand factor gene. Animal Genetics, 25(2), 122. 10.1111/j.1365-2052.1994.tb00094.x 8010530

[ece32949-bib-0078] Sundqvist, A.‐K. , et al. (2006). Unequal contribution of sexes in the origin of dog breeds. Genetics, 172(2), 1121–1128. 16219789 10.1534/genetics.105.042358 PMC1456210

[ece32949-bib-0079] Torres, R. T. , & Fonseca, C. (2016). Perspectives on the Iberian wolf in Portugal: Population trends and conservation threats. Biodiversity and Conservation, 25(3), 411–425.

[ece32949-bib-0080] Valière, N. , et al. (2003). Long‐distance wolf recolonization of France and Switzerland inferred from non‐invasive genetic sampling over a period of 10 years. Animal Conservation, 6(1), 83–92.

[ece32949-bib-0081] Vilà, C. , & Wayne, R. K. (1999). Hybridization between Wolves and Dogs. Conservation Biology, 13(1), 195–198.

[ece32949-bib-0082] Vilà, C. , et al. (1997). Multiple and ancient origins of the domestic dog. Science, 276(5319), 1687–1689. 9180076 10.1126/science.276.5319.1687

[ece32949-bib-0083] Vilà, C. , et al. (1999). Mitochondrial DNA phylogeography and population history of the grey wolf canis lupus. Molecular Ecology, 8(12), 2089–2103. 10632860 10.1046/j.1365-294x.1999.00825.x

[ece32949-bib-0084] Vilà, C. , et al. (2003). Combined use of maternal, paternal and bi‐parental genetic markers for the identification of wolf–dog hybrids. Heredity, 90(1), 17–24. 12522421 10.1038/sj.hdy.6800175

[ece32949-bib-0085] Walsh, P. , Metzger, D. , & Higuchi, R. (1991). Chelex 100 as a medium for simple extraction of DNA for PCR‐based typing from forensic material. BioTechniques, 10(4), 506–513. 1867860

