# Supplementary material for: New insights into the genetic composition and phylogenetic relationship of wolves and dogs in the Iberian Peninsula
Source: Ecol Evol. 2017 May 11;7(12):4404–18. doi: 10.1002/ece3.2949 (PMC5478058; doi:10.1002/ece3.2949)
Supplement: Supplementary file 1 [file ECE3-7-4404-s001.docx]

**Supplementary Materials for**

**New insights into the genetic composition and phylogenetic relationship of wolves and dogs in the Iberian Peninsula**

**Genetic markers**

## Autosomal STR genotyping

PCR reactions were prepared in multiplex reactions using the Qiagen multiplex kit and according to manufacturer. PCR products were subject to electrophoresis on an ABI 310 capillary sequencer (Applied Biosystems, Foster City, CA, USA) and alleles were scored relative to the internal size standard Genescan 350 Rox (Applied Biosystems), using GeneScan v3.7.

**Y chromosome SNPs**

PCR Amplification

Loci Ydog_28, Ydog_B and Ydog_G, were PCR amplified in a Primus 96 Plus thermocycler (MWGAC Biotech) using a touchdown procedure consisting of an initial denaturation step at 94 °C (3 min), 11 cycles of denaturation at 94 ºC (30 s), annealing at 53 ºC (45 s) and extension at 72 ºC (1 min), followed by 24 cycles of 94 ºC (30 s), 48 ºC (45 s) and 72 ºC (1 min) and a final extension step at 72 ºC for 20 minutes. For the remaining loci, PCR amplifications were performed in a iCycler thermocycler (Biorad), using a first denaturation step at 95 °C (30 s), followed by 35 cycles of 95 ºC (30 s), 50 ºC (45 s) and 72 ºC (30 s) and a final extension step at 72 ºC for 30 minutes. PCR amplicons were monitored on 1.5% (w/v) agarose gel and further treated with ExoSapIT (Amershan Pharmacia Biotech) by incubating 5 μL of PCR product with 2 μL of ExoSapIT for 60 minutes at 37 °C, followed by an enzyme inactivation step at 80 °C (15 min) to remove unincorporated primers and dNTPs. The final reaction volume was 10 μL containing 3 μL of the pooled first-step PCR amplicons, 5 μL of SNaPshot ready reaction premix containing fluorescent dideoxy nucleotides and probe primers. The reaction was performed following the manufacturer’s instructions. Capillary electrophoresis was in an ABI PRISM 3130 genetic analyzer (Applied Biosystems, Foster City, CA, USA) using the GeneScan-120 LIZ ladder. Data were analysed using GeneMapper v4.0 software (Applied Biosystems, Foster City, CA, USA).

**Y chromosome microsatellites**

PCR Amplification

PCR reactions were carried out in 12 µL using 1x Qiagen multiplex PCR master mix, 0.5x Q-solution, 0.2 μM of final primer concentration and template DNA (25-50 ng). Amplification reactions were performed in a GeneAmp PCR System 2700 (Perkin Elmer) thermal cycler with an initial activation step at 95 ºC (15 min) followed by a 3-step cycling of denaturation at 94 ºC (30s), annealing at 60 ºC (90s) and extension at 72 ºC (60s) for 30 cycles with a final extension step at 60 ºC (30 min). PCR products were separated by electrophoresis, analyzed on an automated fluorescence-based ABI PRISM 3130 Genetic Analyzer using the GeneScan-350 ROX internal size standard for allele sizing (Applied Biosystems, Foster City, CA, USA), and the data were analysed using the GeneMapper v4.0 software. Y chromosome microsatellite genotyping errors checking, was performed by independent PCR amplifications and allele readings for multiple times.

**Mitochondrial DNA**

**PCR amplification and sequencing**

PCR reactions consisted of 25 pmol of each primer, 1 mM dNTPs, 0.01 mg of BSA, and 1 unit of Taq DNA polymerase, appropriate reaction buffer, 2.5 mM MgCl_2_ and 1-2 µL of template DNA in a total reaction volume of 25 µL. PCR reactions were performed in a Primus 96 Plus thermocycler (MWGAC Biotech) using a touchdown procedure consisting of an initial denaturation step at 95 °C (3 min), 11 cycles of denaturation at 94 ºC (30 s), annealing starting at 53 ºC (45 s) and decreasing 0.5 ºC per cycle, and extension at 72 ºC (1 min), followed by 24 cycles of 94 ºC (30 s), 48 ºC (45 s) and 72 ºC (1 min) and a final extension step at 72 ºC for 20 minutes. PCR amplicons were monitored on 1.5% (w/v) agarose gel and purified by treatment with ExoSapIT (Amersham Pharmacia Biotech) as mentioned above. Purified PCR products were cycle-sequenced in both directions with the PCR amplification primers.

**SUPPLEMENTARY TABLES AND FIGURES**

[**Table S1**](http://www.pubmedcentral.nih.gov/articlerender.fcgi?artid=1924843&rendertype=table&id=T1). Newly designed PCR forward (F) and reverse (R) primer sequences (5'→3') to amplify Y chromosome fragments described by (Natanaelsson et al., 2006). GenBank accession numbers of reference sequences are also indicated.

| **PCR primer sequences** | **PCR product size (bp)** | **Y chromosome fragment/**  **Accession number** |
| --- | --- | --- |
| F: TTCTACTCTCCTTTTACACATTTTC  R: GTGATACTGAAGAATCAAGGC | 171 | Ydog_20/DQ973638 |
| F: CAGAAGAGTAAGTAAGGTGC  R: CCTTAGGGATGGGGCCTG | 180 | Ydog_21/DQ973639 |
| F: TTCAGTTCTGTTCACACCAAAAC  R: AGAGGACAAAAAGCATATC | 422 | Ydog_28/DQ973642 |
| F: CATTTGAAATTTGAACATTTGAAATTTGAA  R: GTGACTCCTGTCTAAGCAAGG | 244 | Ydog_29/DQ973635 |
| F: TAGGCAGAGGGAGAAGGAGG  R: TAAAATTAGATTCTTGGGGTGGCC | 130 | Ydog_30/DQ973636 |
| F: AGCGCCGGAGCCGGCGG  R: GCACTGCTGACTGGGTG | 190 | Ydog_B/DQ973631 |
| F: GGAATCCCTTTGCAAAGG  R: GGCAAAAGTCCGGGTGTCCTAC | 492 | Ydog_G/DQ973626 |
| F: CTATTTTAGCTGGCCATTTGCTTGG  R: GAACTGTAATCACTGTGGCCTC | 180 | Ydog_N/DQ973627 |

**Table S2 -** Extension primer sequences designed to detect Y chromosome SNPs.

| **Y chromosome**  **Fragment** | **Extension primers (5’-3’)** | **Annealing strand** | **Primer Length**  (bases) | **Expected substitutions in dogs – Nucleotide Position ^*^** |
| --- | --- | --- | --- | --- |
| Ydog_20 | AAATACTACTAGGATGAAATATAAC | Anti-sense | 25 | A/G - 35 |
| Ydog_21 | CTTGGGGCACCTGGGTAGCTCACTCA | Sense | 26 | A/G - 417 |
| Ydog_28 | A - TCTAAATCAGCATCTTTTAAAAGCTCGT  B - GGTAACTTACTGTGAGAAAGAATAAAGTATTTA  C - CACTTTACACATTGTTAGAAGATTGTTTCTGTACATT | Sense  Anti-sense  Sense | 28  33  37 | A/G - 599  A/C - 619  G/A - 873 |
| Ydog_29 | TTTTCTATTTTATTTCAAACTCAAATTAGGAATATCTTTT | Anti-sense | 40 | A/T - 56 |
| Ydog_30 | AAAGCGGCACTAAACCGCTGAGCCACC | Sense | 27 | C/T - 655 |
| Ydog_B (part2) | gcagcccacagtttttagcttctccaaaatgac | Sense | 33 | C/T - 225 |
| Ydog_G (part1) | A - TTGCAAAGGACAGGTTCATTGGTCTCTCAGCTACT  B – TACATATGTAAATTATATCTTCTTTGGTACTGTATATTATCTCTC | Sense  Sense | 35  45 | T/C – 66  C/A – 146 |
| Ydog_N | AACCAAATCAAACCAAACATAACC | Anti-sense | 24 | C/G - 608 |

*Positions are denoted relative to GenBank sequences (DQ973626 – DQ973805)

**Table S3** – Primers sequences used for Y-STRs genotyping. MS41A and B loci are discernible using the indicated primer pair.

| **Locus Name** | **Forward (5’-3’)** | **Reverse (5’-3’)** |
| --- | --- | --- |
| 990-35 | **FAM**-CCATCCGCAGAACAGGTATT | GGGCCGCTATTTTAGGTGAT |
| MS34TTR | **FAM**-AAGCATTCTCTTCCAGTTGGTC | TGAACCATCATTGTGAATATGAA |
| MS41B | TCCTCTAATTTTCCCCTCTC | **HEX**-CTGCTCGACCCTCTTCTCTG |
| MS41A | *The same sequence as for locus MS41B* | *The same sequence as for locus MS41B* |

FAM=6-FAM^TM^ Fluorescein, HEX=hexachlorofluorescein phosphoramidite

**Table S4A** – Y chromosome SNP haplotypes and their distribution in each *Canis* population. A total of 563 individuals, including worldwide domestic dogs and wolves were considered, combining results from this study and others (Brown *et al.* 2011 and Ding *et al.* 2012).

| . |  | **Ydog fragment and associated SNP** | | **20** | **21** | **28A** | **28B** | **28C** | **29** | **30** |  | **G1A** | **G1B** | **N** |  |  |  | | |
| --- | --- | --- | --- | --- | --- | --- | --- | --- | --- | --- | --- | --- | --- | --- | --- | --- | --- | --- | --- |
|  |  |  |  |  |  |  |  |  |  |  | **B** |  |  |  |  |  |  | | |
| **Haplotype Code** | **Haplotype designation following Brown *et al*. 2011** | **Haplotype designation following Ding *et al*. 2011** | **Reference haplotype:** | **A** | **G** | **A** | **A** | **G** | **A** | **C** | **C** | **T** | **C** | **C** | **Count** | ***Canis* population** | **Source** | | |
| ***YSNP H1*** | 1/2/3/4 | H1/H2/H3/H4/H11/H12/H13/H14/H17/H20/H21/H24/H31 |  | . | . | . | . | . | . | . | . | . | . | . | **278** | Domestic dog | Brown *et al*. 2011; Ding *et al*. 2012; this study | | |
| ***YSNP H5*** | 5 | H5/H22 |  | . | . | . | . | . | T | . | . | . | . | . | **17** | Domestic dog | Brown *et al*. 2011; Ding *et al*. 2012 | | |
| ***YSNP H6*** | 6 | H6/H15/H16/H19 |  | G | . | . | . | . | . | . | . | . | . | G | **68** | Domestic dog | Brown *et al*. 2011; Ding *et al.* 2012 | | |
| ***YSNP H7*** | 7 | H7 |  | . | . | G | C | A | . | . | . | C | A | . | **13** | Domestic dog | Brown *et al.* 2011; Ding *et al.* 2012 | | |
| ***YSNP H23*** | - | H23*/H25 |  | . | . | G | C | A | . | . | . | C/T | A | . | **20** | Domestic dog; Wolf | Ding *et al.* 2012 | | |
| ***YSNP H8*** | 8 | H8 |  | . | . | G | C | A | . | T | . | . | A | . | **21** | Domestic dog | Brown et al. 2011; Ding *et al.* 2012 | | |
| ***YSNP H9*** | 9 | H9/H18 |  | . | A | G | C | . | . | . | T | . | A | . | **6** | Domestic dog; Iberian wolf | Brown *et al.* 2011; Ding *et al.* 2012; this study | | |
| ***YSNP 10B*** | 10 |  |  | . | A | G | C | . | . | . | . | . | A | . | **8** | Domestic dog; Wolf | Brown *et al*. 2011 | | |
| ***YSNP H10*** | 11 | H10/H23a |  | . | . | G | C | A | . | . | . | . | A | . | **95** | Domestic dog; Wolf | Brown *et al.* 2011; Ding *et al.* 2012 | | |
| ***YSNP H27*** | 12 | H27a,b |  | . | . | G | C | . | . | . | . | . | A | . | **11** | Wolf | Brown *et al.* 2011; Ding *et al.* 2012 | | |
| ***YSNP H26*** | - | H26 |  | . | . | . | . | . | . | . | . | . | . | G | **1** | American wolf | Ding *et al.* 2012 | | |
| ***YSNP H32*** | - | - |  | . | . | G | C | . | . | . | T | . | A | . | **23** | Iberian Wolf | this study | | |
| ***YSNP H33*** | - | - |  | . | A | G | C | A | . | . | T | . | A | . | **1** | Iberian Wolf | | this study |  |
| ***YSNP H34*** | - | - |  | . | . | G | C | A | . | . | T | . | A | . | **1** | Iberian Wolf | this study | | |

**Table S4B** – Y chromosome STR haplotypes and their distribution in each of the dog breeds or *Canis* population studied. A total of 108 male individuals, including both domestic dogs and wolves were considered. Breed nomenclature as in Figure 1.

| **Number of repeats per loci:**  Loci 990-35/MS41A/MS41B/MS34TTR | **Haplotype code** | **Dog breed(n) or *Canis* population(n)** | **Total Count** | ***Canis* population** |
| --- | --- | --- | --- | --- |
| 12/16/24/12 | YSTR_H01 | PWD(2) | 2 | Domestic Dog |
| 12/17/25/12 | YSTR_H02 | IbVillDog(2); Aidi(1); PWD(2); ACD(5); SM(2); PWH(2); EMD(1); TunisiaDog(1) | 16 | Domestic Dog |
| 12/17/25/14 | YSTR_H03 | IbVillDog(1) | 1 | Domestic Dog |
| 12/18/26/12 | YSTR_H04 | Aidi(1); PWH(4); Sloughi(2) | 7 | Domestic Dog |
| 12/18/27/11 | YSTR_H05 | TM(1) | 1 | Domestic Dog |
| 12/18/27/12 | YSTR_H06 | AM(1) | 1 | Domestic Dog |
| 12/19/26/10 | YSTR_H07 | CLWD(1) | 1 | Domestic Dog |
| 12/19/26/13 | YSTR_H08 | EMD(1) | 1 | Domestic Dog |
| 12/19/27/11 | YSTR_H09 | IbWolf(2); SM(1); AM(1); EMD(1) | 5 | Iberian Wolf; Domestic Dog |
| 12/19/27/12 | YSTR_H10 | IbWolf(1); IbVillDog(4); Aidi(1); ACD(2): TM(3); CLWD(4); SP(3); PP(3); AM(2); PSD(2); EMD(1) | 26 | Iberian Wolf; Domestic Dog |
| 12/19/27/13 | YSTR_H11 | PWH(1); Sloughi(1) | 2 | Domestic Dog |
| 12/20/26/12 | YSTR_H12 | Sloughi(1) | 1 | Domestic Dog |
| 12/20/27/12 | YSTR_H13 | Aidi(1) | 1 | Domestic Dog |
| 12/20/27/13 | YSTR_H14 | EMD(1) | 1 | Domestic Dog |
| 12/20/28/11 | YSTR_H15 | IbWolf(3) | 3 | Iberian Wolf |
| 12/20/28/12 | YSTR_H16 | SP(1); EMD(1) | 2 | Domestic Dog |
| 12/20/29/12 | YSTR_H17 | AM(1) | 1 | Domestic Dog |
| 12/21/26/12 | YSTR_H18 | AM(1) | 1 | Domestic Dog |
| 12/21/28/12 | YSTR_H19 | PWD(1) | 1 | Domestic Dog |
| 13/17/25/11 | YSTR_H20 | IbVillDog(1) | 1 | Domestic Dog |
| 13/18/26/11 | YSTR_H21 | CLWD(1); AM(1); PSD(1); EMD(2) | 5 | Domestic Dog |
| 13/18/27/11 | YSTR_H22 | Aidi(1) | 1 | Domestic Dog |
| 13/19/26/10 | YSTR_H23 | CLWD(1) | 1 | Domestic Dog |
| 13/19/27/12 | YSTR_H24 | IbWolf(1); PWD(1); CLWD(1); AM(1) | 4 | Iberian Wolf; Domestic Dog |
| 13/20/25/13 | YSTR_H25 | EMD(1) | 1 | Domestic Dog |
| 13/20/27/12 | YSTR_H26 | IbWolf(14) | 14 | Iberian Wolf |
| 13/20/28/11 | YSTR_H27 | Aidi(1) | 1 | Domestic Dog |
| 13/20/29/12 | YSTR_H28 | IbWolf(3) | 3 | Iberian Wolf |
| 13/21/29/12 | YSTR_H29 | IbWolf(3) | 3 | Iberian Wolf |

**Table S5 –** Samples for which mitochondrial and/or nuclear molecular data was generated: 196 dogs (86 males, 110 females) from Iberia and North Africa, and 56 Iberian wolves (44 males, 11 females, 1 unknown gender). Designations of breeds/populations are indicated for dog samples, and geographic origin is indicated for Iberian wolf samples. For the Iberian wolf samples provided by the Portuguese sample tissue bank (SMLM, ICNF), original tissue bank codes are indicated in parenthesis.

IbWolf- Iberian wolf; IbDog - Iberian dog; AfrDog - African dog; mtDNA - haplotypes for 420 bp of mitochondrial DNA control region I followed by GenBank accession number; Y-markers - sample typed for dog specific Y chromosome SNPs (n=11) and Y chromosome STRs loci (n=4); m - male; f - female; ? - unknown gender.

*For the wolf samples sequenced, longer haplotypes (420 bp) that collapsed to the same haplotype when trimmed to 230 bp were given the same root label followed by a letter *e.g.,* wH-1A, wH-1B and wH-1C are indistinct when trimmed to 230 bp.; dog haplotype codes as in the original publication Pires *et al.* (2006).

** not different from Björnerfeldt *et al.* (2006) GenBank DQ480505.1 (complete mtDNA genome, Spain isolate 1)

*** not different from Pires *et al.* (2006) GenBank AY706485 and AY706523 (887 bp, dog haplotypes H10 and H48, respectively)

| **Sample Code** | **Sex** | **Y-markers** | **mtDNA** | **Dog breed / Wolf origin** |
| --- | --- | --- | --- | --- |
| IbDog-1 | f |  | H09, AY706484 | Portuguese Water dog |
| IbDog-2 | f |  | H09, AY706484 | Portuguese Water dog |
| IbDog-3 | m | YSTR_H19; YSNP_H1 | H10, AY706485 | Portuguese Water dog |
| IbDog-4 | f |  | H11, AY706486 | Portuguese Water dog |
| IbDog-5 | m | YSTR_H01; YSNP_H1 | H07, AY706482 | Portuguese Water dog |
| IbDog-6 | m | YSTR_H01; YSNP_H1 | H07, AY706482 | Portuguese Water dog |
| IbDog-7 | f |  | H07, AY706482 | Portuguese Water dog |
| IbDog-8 | m | YSTR_H02; YSNP_H1 | H11, AY706486 | Portuguese Water dog |
| IbDog-9 | m | YSTR_H24; YSNP_H1 | H12, AY706487 | Portuguese Water dog |
| IbDog-10 | m | YSTR_H02; YSNP_H1 | H11, AY706486 | Portuguese Water dog |
| IbDog-11 | f |  | H11, AY706486 | Portuguese Water dog |
| IbDog-12 | f |  | H13, AY706488 | Portuguese village dog |
| IbDog-13 | f |  | H14, AY706489 | Portuguese village dog |
| IbDog-14 | f |  | H15, AY706490 | Portuguese village dog |
| IbDog-15 | m | YSTR_H20; YSNP_H1 | H16, AY706491 | Portuguese village dog |
| IbDog-16 | f |  | H17, AY706492 | Portuguese village dog |
| IbDog-17 | f |  | H13, AY706488 | Portuguese village dog |
| IbDog-18 | f |  | H14, AY706489 | Portuguese village dog |
| IbDog-19 | f |  | H18, AY706493 | Portuguese village dog |
| IbDog-20 | f |  | H19, AY706494 | Portuguese village dog |
| IbDog-21 | f |  | H20, AY706495 | Portuguese village dog |
| IbDog-22 | f |  | H21, AY706496 | Portuguese village dog |
| IbDog-23 | f |  | H22, AY706497 | Portuguese village dog |
| IbDog-24 | m | YSTR_H02; YSNP_H1 |  | Portuguese village dog |
| IbDog-25 | m | YSTR_H10; YSNP_H1  ; YSNP_H1 |  | Portuguese village dog |
| IbDog-26 | m | YSTR_H10; YSNP_H1 |  | Portuguese village dog |
| IbDog-27 | m | YSTR_H03; YSNP_H1  ; YSNP_H1 |  | Portuguese village dog |
| IbDog-28 | m | YSTR_H10; YSNP_H1 |  | Portuguese village dog |
| IbDog-29 | m | YSTR_H02; YSNP_H1 |  | Portuguese village dog |
| IbDog-30 | m | YSTR_H10; YSNP_H1 |  | Portuguese village dog |
| IbDog-31 | f |  | H21, AY706496 | Azores Cattle dog |
| IbDog-32 | f |  | H21, AY706496 | Azores Cattle dog |
| IbDog-33 | f |  | H21, AY706496 | Azores Cattle dog |
| IbDog-34 | f |  | H23, AY706498 | Azores Cattle dog |
| IbDog-35 | f |  | H21, AY706496 | Azores Cattle dog |
| IbDog-36 | f |  | H23, AY706498 | Azores Cattle dog |
| IbDog-37 | f |  | H23, AY706498 | Azores Cattle dog |
| IbDog-38 | f |  | H21, AY706496 | Azores Cattle dog |
| IbDog-39 | f |  | H21, AY706496 | Azores Cattle dog |
| IbDog-40 | f |  | H24, AY706499 | Azores Cattle dog |
| IbDog-41 | m | YSTR_H02; YSNP_H1 | H23, AY706498 | Azores Cattle dog |
| IbDog-42 | f |  | H21, AY706496 | Azores Cattle dog |
| IbDog-43 | f |  | H23, AY706498 | Azores Cattle dog |
| IbDog-44 | f |  | H17, AY706492 | Azores Cattle dog |
| IbDog-45 | m | YSTR_H10; YSNP_H1 |  | Azores Cattle dog |
| IbDog-46 | m | YSTR_H10; YSNP_H1 |  | Azores Cattle dog |
| IbDog-47 | m | YSTR_H02; YSNP_H1  ; YSNP_H1 |  | Azores Cattle dog |
| IbDog-48 | m | YSTR_H02; YSNP_H1 |  | Azores Cattle dog |
| IbDog-49 | m | YSTR_H02; YSNP_H1 |  | Azores Cattle dog |
| IbDog-50 | m | YSTR_H02; YSNP_H1 |  | Azores Cattle dog |
| IbDog-51 | m | YSTR_H10; YSNP_H1 |  | Transmontano Mastiff |
| IbDog-52 | m | YSTR_H05; YSNP_H1 |  | Transmontano Mastiff |
| IbDog-53 | m | YSTR_H10; YSNP_H1 |  | Transmontano Mastiff |
| IbDog-54 | m | YSTR_H10; YSNP_H1 |  | Transmontano Mastiff |
| IbDog-55 | m | YSTR_H10; YSNP_H1 | H25, AY706500 | Castro Laboreiro Watchdog |
| IbDog-56 | f |  | H25, AY706500 | Castro Laboreiro Watchdog |
| IbDog-57 | f |  | H25, AY706500 | Castro Laboreiro Watchdog |
| IbDog-58 | m | YSTR_H10; YSNP_H1 | H25, AY706500 | Castro Laboreiro Watchdog |
| IbDog-59 | m | YSTR_H07; YSNP_H1 | H25, AY706500 | Castro Laboreiro Watchdog |
| IbDog-60 | f |  | H25, AY706500 | Castro Laboreiro Watchdog |
| IbDog-61 | f |  | H25, AY706500 | Castro Laboreiro Watchdog |
| IbDog-62 | m | YSTR_H24; YSNP_H1 | H25, AY706500 | Castro Laboreiro Watchdog |
| IbDog-63 | m | YSTR_H23; YSNP_H1 |  | Castro Laboreiro Watchdog |
| IbDog-64 | m | YSTR_H21; YSNP_H1  √ |  | Castro Laboreiro Watchdog |
| IbDog-65 | m | YSTR_H10; YSNP_H1 |  | Castro Laboreiro Watchdog |
| IbDog-66 | f |  | H25, AY706500 | Castro Laboreiro Watchdog |
| IbDog-67 | f |  | H25, AY706500 | Castro Laboreiro Watchdog |
| IbDog-68 | f |  | H25, AY706500 | Castro Laboreiro Watchdog |
| IbDog-69 | m | YSTR_H10; YSNP_H1 | H25, AY706500 | Castro Laboreiro Watchdog |
| IbDog-70 | f |  | H25, AY706500 | Castro Laboreiro Watchdog |
| IbDog-71 | f |  | H25, AY706500 | Castro Laboreiro Watchdog |
| IbDog-72 | f |  | H10, AY706485 | Spanish Mastiff |
| IbDog-73 | m | YSTR_H10; YSNP_H1 | H24, AY706499 | Spanish Mastiff |
| IbDog-74 | f |  | H26, AY706501 | Spanish Mastiff |
| IbDog-75 | m | YSTR_H02; YSNP_H1 | H04, AY706479 | Spanish Mastiff |
| IbDog-76 | m | YSTR_H10; YSNP_H1 | H04, AY706479 | Spanish Mastiff |
| IbDog-77 | m | YSTR_H10; YSNP_H1 | H24, AY706499 | Spanish Mastiff |
| IbDog-78 | m | YSTR_H16; YSNP_H1 | H24, AY706499 | Spanish Mastiff |
| IbDog-79 | m | YSTR_H09; YSNP_H1 | H27, AY706502 | Spanish Mastiff |
| IbDog-80 | m | YSTR_H02; YSNP_H1 | H13, AY706488 | Spanish Mastiff |
| IbDog-81 | f |  | H24, AY706499 | Spanish Mastiff |
| IbDog-82 | f |  | H07, AY706482 | Portuguese Pointer |
| IbDog-83 | f |  | H07, AY706482 | Portuguese Pointer |
| IbDog-84 | f |  | H07, AY706482 | Portuguese Pointer |
| IbDog-85 | f |  | H07, AY706482 | Portuguese Pointer |
| IbDog-86 | f |  | H07, AY706482 | Portuguese Pointer |
| IbDog-87 | m | YSTR_H10; YSNP_H1 | H28, AY706503 | Portuguese Pointer |
| IbDog-88 | f |  | H28, AY706503 | Portuguese Pointer |
| IbDog-89 | m | YSTR_H10; YSNP_H1 | H28, AY706503 | Portuguese Pointer |
| IbDog-90 | m | YSTR_H10; YSNP_H1 | H07, AY706482 | Portuguese Pointer |
| IbDog-91 | f |  | H29, AY706504 | Portuguese Warren Hound |
| IbDog-92 | f |  | H10, AY706485 | Portuguese Warren Hound |
| IbDog-93 | f |  | H30, AY706505 | Portuguese Warren Hound |
| IbDog-94 | f |  | H10, AY706485 | Portuguese Warren Hound |
| IbDog-95 | f |  | H17, AY706492 | Portuguese Warren Hound |
| IbDog-96 | f |  | H10, AY706485 | Portuguese Warren Hound |
| IbDog-97 | f |  | H10, AY706485 | Portuguese Warren Hound |
| IbDog-98 | m | YSTR_H11; YSNP_H1 | H07, AY706482 | Portuguese Warren Hound |
| IbDog-99 | f |  | H07, AY706482 | Portuguese Warren Hound |
| IbDog-100 | f |  | H28, AY706503 | Portuguese Warren Hound |
| IbDog-101 | f |  | H17, AY706492 | Portuguese Warren Hound |
| IbDog-102 | m | YSTR_H02; YSNP_H1 | H13, AY706488 | Portuguese Warren Hound |
| IbDog-103 | f |  | H17, AY706492 | Portuguese Warren Hound |
| IbDog-104 | f |  | H31, AY706506 | Portuguese Warren Hound |
| IbDog-105 | f |  | H31, AY706506 | Portuguese Warren Hound |
| IbDog-106 | f |  | H10, AY706485 | Portuguese Warren Hound |
| IbDog-107 | f |  | H29, AY706504 | Portuguese Warren Hound |
| IbDog-108 | f |  | H32, AY706507 | Portuguese Warren Hound |
| IbDog-109 | f |  | H33, AY706508 | Portuguese Warren Hound |
| IbDog-110 | m | YSTR_H04; YSNP_H1 | H21, AY706496 | Portuguese Warren Hound |
| IbDog-111 | m | YSTR_H04; YSNP_H1 | H34, AY706509 | Portuguese Warren Hound |
| IbDog-112 | f |  | H17, AY706492 | Portuguese Warren Hound |
| IbDog-113 | f |  | H35, AY706510 | Portuguese Warren Hound |
| IbDog-114 | f |  | H10, AY706485 | Portuguese Warren Hound |
| IbDog-115 | f |  | H21, AY706496 | Portuguese Warren Hound |
| IbDog-116 | m | YSTR_H02; YSNP_H1 | H21, AY706496 | Portuguese Warren Hound |
| IbDog-117 | f |  | H10, AY706485 | Portuguese Warren Hound |
| IbDog-118 | m | YSTR_H04; YSNP_H1 |  | Portuguese Warren Hound |
| IbDog-119 | m | YSTR_H04; YSNP_H1 |  | Portuguese Warren Hound |
| IbDog-120 | m | YSTR_H24; YSNP_H1 | H05, AY706480 | Alentejo Mastiff |
| IbDog-121 | m | YSTR_H21; YSNP_H1 | H28, AY706503 | Alentejo Mastiff |
| IbDog-122 | f |  | H10, AY706485 | Alentejo Mastiff |
| IbDog-123 | f |  | H05, AY706480 | Alentejo Mastiff |
| IbDog-124 | f |  | H10, AY706485 | Alentejo Mastiff |
| IbDog-125 | f |  | H33, AY706508 | Alentejo Mastiff |
| IbDog-126 | f |  | H28, AY706503 | Alentejo Mastiff |
| IbDog-127 | f |  | H23, AY706498 | Alentejo Mastiff |
| IbDog-128 | m | YSTR_H18; YSNP_H1 | H36, AY706511 | Alentejo Mastiff |
| IbDog-129 | m | YSTR_H06; YSNP_H1 | H37, AY706512 | Alentejo Mastiff |
| IbDog-130 | f |  | H05, AY706480 | Alentejo Mastiff |
| IbDog-131 | m | YSTR_H10; YSNP_H1 | H36, AY706511 | Alentejo Mastiff |
| IbDog-132 | f |  | H38, AY706513 | Alentejo Mastiff |
| IbDog-133 | m | YSTR_H10; YSNP_H1 | H37, AY706512 | Alentejo Mastiff |
| IbDog-134 | f |  | H23, AY706498 | Alentejo Mastiff |
| IbDog-135 | m | YSTR_H09; YSNP_H1 |  | Alentejo Mastiff |
| IbDog-136 | m | YSTR_H28; YSNP_H1 |  | Alentejo Mastiff |
| IbDog-137 | f |  | H10, AY706485 | Portuguese Sheepdog |
| IbDog-138 | f |  | H10, AY706485 | Portuguese Sheepdog |
| IbDog-139 | m | YSTR_H10; YSNP_H1 | H10, AY706485 | Portuguese Sheepdog |
| IbDog-140 | f |  | H10, AY706485 | Portuguese Sheepdog |
| IbDog-141 | m | YSTR_H21; YSNP_H1 | H10, AY706485 | Portuguese Sheepdog |
| IbDog-142 | f |  | H10, AY706485 | Portuguese Sheepdog |
| IbDog-143 | f |  | H39, AY706514 | Portuguese Sheepdog |
| IbDog-144 | f |  | H10, AY706485 | Portuguese Sheepdog |
| IbDog-145 | m | YSTR_H10; YSNP_H1 |  | Portuguese Sheepdog |
| IbDog-146 | f |  | H40, AY706515 | Estrela Mountain dog |
| IbDog-147 | f |  | H05, AY706480 | Estrela Mountain dog |
| IbDog-148 | m | YSTR_H08; YSNP_H1 | H05, AY706480 | Estrela Mountain dog |
| IbDog-149 | f |  | H33, AY706508 | Estrela Mountain dog |
| IbDog-150 | f |  | H40, AY706515 | Estrela Mountain dog |
| IbDog-151 | f |  | H17, AY706492 | Estrela Mountain dog |
| IbDog-152 | f |  | H40, AY706515 | Estrela Mountain dog |
| IbDog-153 | f |  | H17, AY706492 | Estrela Mountain dog |
| IbDog-154 | f |  | H40, AY706515 | Estrela Mountain dog |
| IbDog-155 | f |  | H13, AY706488 | Estrela Mountain dog |
| IbDog-156 | f |  | H41, AY706516 | Estrela Mountain dog |
| IbDog-157 | f |  | H07, AY706482 | Estrela Mountain dog |
| IbDog-158 | m | YSTR_H09; YSNP_H1 | H42, AY706517 | Estrela Mountain dog |
| IbDog-159 | f |  | H17, AY706492 | Estrela Mountain dog |
| IbDog-160 | f |  | H13, AY706488 | Estrela Mountain dog |
| IbDog-161 | m | YSTR_H14; YSNP_H1 |  | Estrela Mountain dog |
| IbDog-162 | m | YSTR_H02; YSNP_H1 |  | Estrela Mountain dog |
| IbDog-163 | m | YSTR_H16; YSNP_H1 |  | Estrela Mountain dog |
| IbDog-164 | m | YSTR_H10; YSNP_H1 |  | Estrela Mountain dog |
| IbDog-165 | m | YSTR_H21; YSNP_H1 |  | Estrela Mountain dog |
| IbDog-166 | m | YSTR_H25; YSNP_H1 |  | Estrela Mountain dog |
| IbDog-167 | m | YSTR_H21; YSNP_H1 |  | Estrela Mountain dog |
| AfrDog-1 | f |  | H03, AY706478 | Aidi |
| AfrDog-2 | f |  | H04, AY706479 | Aidi |
| AfrDog-3 | f |  | H01, AY706476 | Aidi |
| AfrDog-4 | m | YSTR_H27; YSNP_H1 | H02, AY706477 | Aidi |
| AfrDog-5 | m | YSTR_H02; YSNP_H1 | H01, AY706476 | Aidi |
| AfrDog-6 | m | YSTR_H22; YSNP_H1 | H05, AY706480 | Aidi |
| AfrDog-7 | f |  | H06, AY706481 | Aidi |
| AfrDog-8 | m | YSTR_H04; YSNP_H1 | H07, AY706482 | Aidi |
| AfrDog-9 | m | YSTR_H10; YSNP_H1 | H08, AY706483 | Aidi |
| AfrDog-10 | m | YSTR_H13; YSNP_H1 | H08, AY706483 | Aidi |
| AfrDog-11 | f |  | H43, AY706518 | Sloughi |
| AfrDog-12 | f |  | H07, AY706482 | Sloughi |
| AfrDog-13 | f |  | H43, AY706518 | Sloughi |
| AfrDog-14 | f |  | H44, AY706519 | Sloughi |
| AfrDog-15 | m | YSTR_H04; YSNP_H1 | H07, AY706482 | Sloughi |
| AfrDog-16 | m | YSTR_H11; YSNP_H1 | H45, AY706520 | Sloughi |
| AfrDog-17 | m | YSTR_H12; YSNP_H1 | H45, AY706520 | Sloughi |
| AfrDog-18 | f |  | H46, AY706521 | Sloughi |
| AfrDog-19 | f |  | H45, AY706520 | Sloughi |
| AfrDog-20 | m | YSTR_H04; YSNP_H1 | H47, AY706522 | Sloughi |
| AfrDog-21 | f |  | H01, AY706476 | Tunisian village dog |
| AfrDog-22 | f |  | H01, AY706476 | Tunisian village dog |
| AfrDog-23 | m | YSTR_H02; YSNP_H1 | H10, AY706485 | Tunisian village dog |
| AfrDog-24 | f |  | H21, AY706496 | Tunisian village dog |
| AfrDog-25 | f |  | H48, AY706523 | Tunisian village dog |
| AfrDog-26 | f |  | H07, AY706482 | Tunisian village dog |
| AfrDog-27 | f |  | H01, AY706476 | Tunisian village dog |
| AfrDog-28 | f |  | H49, AY706524 | Tunisian village dog |
| AfrDog-29 | f |  | H01, AY706476 | Tunisian village dog |
| IbWolf-1 | m | YSTR_H26; YSNP_H32 | wH-1A, ** | Aveiro, Portugal |
| IbWolf-2 (SMLM-19) | m | YSTR_H26;  YSNP_H32 | wH-1A, ** | Braga, Portugal |
| IbWolf-3 | m | YSTR_H28; YSNP_H32 | wH-1A, ** | Braga, Portugal |
| IbWolf-4 | m | YSTR_H29; YSNP_H32 | wH-1A, ** | Braga, Portugal |
| IbWolf-5 | m |  | wH-5, *** | Braga, Portugal |
| IbWolf-6 | f |  | wH-1A, ** | Bragança, Portugal |
| IbWolf-7 | m |  | wH-1C, JX845625 | Bragança, Portugal |
| IbWolf-8 | m | YSTR_H26; YSNP_H32 | wH-1A, ** | Bragança, Portugal |
| IbWolf-9 | m | YSTR_H26; YSNP_H32 | wH-1A, ** | Bragança, Portugal |
| IbWolf-10 | m | YSTR_H26; YSNP_H32 | wH-1A, ** | Bragança, Portugal |
| IbWolf-11 | m | YSTR_H26; YSNP_H32 | wH-1A, ** | Bragança, Portugal |
| IbWolf-12 (SMLM-03) | m | YSTR_H09; YSNP_H32 | wH-2, JX845622 | Bragança, Portugal |
| IbWolf-13 (SMLM-04) | m | YSTR_H10; YSNP_H32 | wH-1A, ** | Bragança, Portugal |
| IbWolf-14 | m | YSTR_H15; YSNP_H34 | wH-1A, ** | Bragança, Portugal |
| IbWolf-15 (SMLM-05) | f |  | wH-1A, ** | Bragança, Portugal |
| IbWolf-16 | m |  | wH-2, JX845622 | Bragança, Portugal |
| IbWolf-17 (SMLM-06) | f |  | wH-1A, ** | Bragança, Portugal |
| IbWolf-18 (SMLM-02) | m | YSTR_H26; YSNP_H32 | wH-1A, ** | Bragança, Portugal |
| IbWolf-19 (SMLM-45) | m | YSTR_H26; YSNP_H32 | wH-1A, ** | Bragança, Portugal |
| IbWolf-20 | f |  | wH-1A, ** | Guarda, Portugal |
| IbWolf-21 (SMLM-70) | m | YSTR_H15; YSNP_H32 | wH-1A, ** | Viana do Castelo, Portugal |
| IbWolf-22 (SMLM-77) | m | YSTR_H26; YSNP_H9 | wH-1A** | Viana do Castelo, Portugal |
| IbWolf-23 | f |  | wH-1A, ** | Viana do Castelo, Portugal |
| IbWolf-24 (SMLM-71) | m | YSTR_H29; YSNP_H9 | wH-1B, JX645624 | Viana do Castelo, Portugal |
| IbWolf-25 (SMLM-26) | f |  | wH-2, JX845622 | Vila Real, Portugal |
| IbWolf-26 | f |  | wH-1A, ** | Vila Real, Portugal |
| IbWolf-27 (SMLM-25) | m | YSTR_H26; YSNP_H32 | wH-4, JX845621 | Vila Real, Portugal |
| IbWolf-28 (SMLM-07) | f |  | wH-1A, ** | Vila Real, Portugal |
| IbWolf-29 (SMLM-48) | m | YSTR_H15; YSNP_H32 | wH-1A, ** | Vila Real, Portugal |
| IbWolf-30 | m |  | wH-1A, ** | Vila Real, Portugal |
| IbWolf-31 | m |  | wH-1A, ** | Vila Real, Portugal |
| IbWolf-32 | m |  | wH-1A, ** | Vila Real, Portugal |
| IbWolf-33 | m |  | wH-1A, ** | Vila Real, Portugal |
| IbWolf-34 | m |  | wH-1A, ** | Vila Real, Portugal |
| IbWolf-35 | m |  | wH-1A, ** | Vila Real, Portugal |
| IbWolf-36 | m |  | wH-1A, ** | Viseu, Portugal |
| IbWolf-37 | m |  | wH-1A, ** | Viseu, Portugal |
| IbWolf-38 (SMLM-22) | m |  | wH-1A, ** | Viseu, Portugal |
| IbWolf-39 (SMLM-42) | m |  | wH-1A, ** | Viseu, Portugal |
| IbWolf-40 | m |  | wH-1A, ** | Viseu, Portugal |
| IbWolf-41 | ? |  | wH-1C | Viseu, Portugal |
| IbWolf-42 | f |  | wH-1A, ** | Viseu, Portugal |
| IbWolf-43 | m | YSTR_H26; YSNP_H32 | wH-1A, ** | Viseu, Portugal |
| IbWolf-44 | f |  | wH-1A, ** | Viseu, Portugal |
| IbWolf-45 | m | YSTR_H29; YSNP_H32 | wH-1A, ** | Viseu, Portugal |
| IbWolf-46 (SMLM-24) | m | YSTR_H26; YSNP_H32 | wH-1A, ** | Viseu, Portugal |
| IbWolf-47 | f |  | wH-1A, ** | Viseu, Portugal |
| IbWolf-48 | m |  | wH-1C, JX845625 | Portugal |
| IbWolf-49 | m |  | wH-1A, ** | Portugal |
| IbWolf-50 | m | YSTR_H28; YSNP_H32 | wH-1A, ** | Portugal |
| IbWolf-51 | m | YSTR_H26; YSNP_H32 | wH-1A, ** | Portugal |
| IbWolf-52 | m | YSTR_H24; YSNP_H32 | wH-3, JX845623 | Galicia, Spain |
| IbWolf-53 | m | YSTR_H09;  YSNP_H33 | wH-1A, ** | Xinzo de Lima, Spain |
| IbWolf-54 (SMLM-01) | m |  | wH-1A, ** | Zamora, Spain |
| IbWolf-55 | m | YSTR_H28; YSNP_H32 | wH-1A, ** | Zamora, Spain |
| IbWolf-56 | m | YSTR_H26; YSNP_H32 | wH-1A, ** | Spain |

**Autosomal STR-based population partitioning**

The population partitioning analysis using the bayesian clustering procedure clearly shows a sharp separation between the dog population, in orange (village dogs and breed dogs, the latter including livestock guard dogs), and the Iberian wolf population, in yellow (Figure S1).


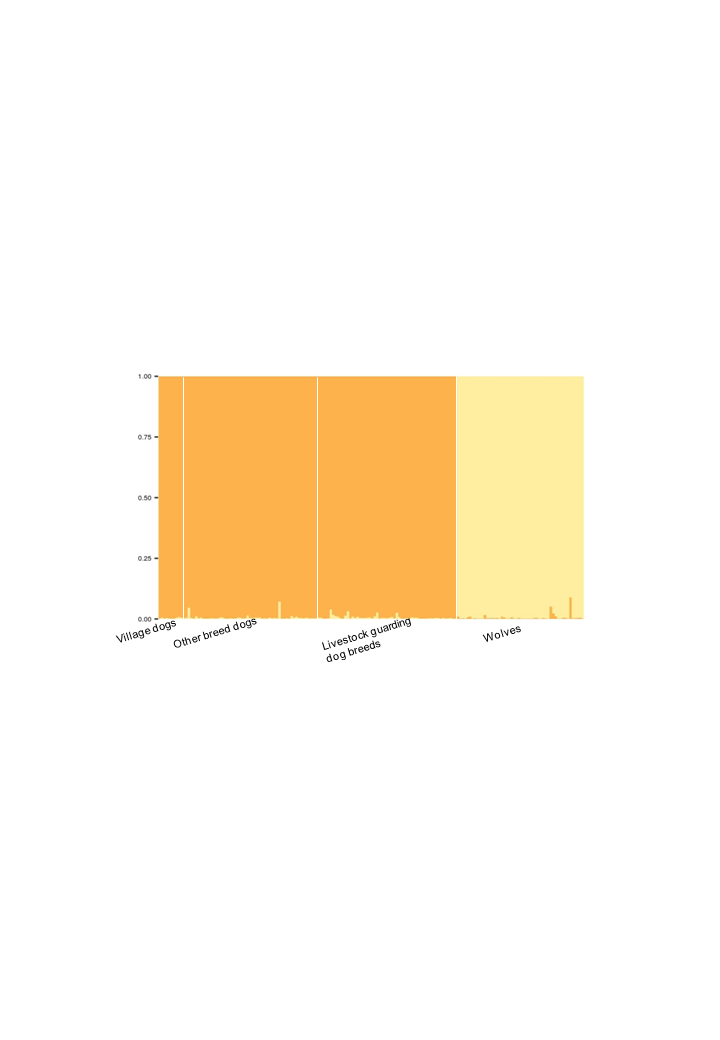


**Figure S1** - Population partitioning suggested by STRUCTURE based on the analyses of 19 microsatellites autosomal loci. Individual assignment using K = 2 clusters. Each individual is represented as a vertical bar sectioned into K coloured segments, whose length is proportional to the likelihood of assignment to the K cluster. Sub-grouping of the dog population includes village dogs, livestock guard dogs and other breed dogs. Those individuals showing a higher percentage of assignment to the opposite cluster (8-8.4%) have an incomplete genotype (missing data for 2 or 3 loci).

**References**

Björnerfeldt, S., Webster, M., & Vilà, C. (2006). Relaxation of selective constraint on dog mitochondrial DNA following domestication. *Genome Research*, *16*(8), 990–994. https://doi.org/10.1101/gr.5117706

Brown, S. K., Pedersen, N. C., Jafarishorijeh, S., Bannasch, D. L., Ahrens, K. D., Wu, J.-T., … Sacks, B. N. (2011). Phylogenetic Distinctiveness of Middle Eastern and Southeast Asian Village Dog Y Chromosomes Illuminates Dog Origins. *PLoS ONE*, *6*(12), e28496. https://doi.org/10.1371/journal.pone.0028496

Ding, Z.-L., Oskarsson, M., Ardalan, A., Angleby, H., Dahlgren, L.-G., Tepeli, C., … Zhang, Y.-P. (2012). Origins of domestic dog in southern East Asia is supported by analysis of Y-chromosome DNA. *Heredity*, *108*(5), 507–14. https://doi.org/10.1038/hdy.2011.114

Natanaelsson, C., Oskarsson, M. C. R., Angleby, H., Lundeberg, J., Kirkness, E., & Savolainen, P. (2006). Dog Y chromosomal DNA sequence: identification, sequencing and SNP discovery. *BMC Genetics*, *7*(1), 45. https://doi.org/10.1186/1471-2156-7-45

Pires, A., Ouragh, L., Kalboussi, M., Matos, J., Petrucci-Fonseca, F., & Bruford, M. W. (2006). Mitochondrial DNA sequence variation in Portuguese native dog breeds: diversity and phylogenetic affinities. *The Journal of Heredity*, *97*(4), 318–30. https://doi.org/10.1093/jhered/esl006
